# Supplementary material for: Structural and mechanistic analysis of a tripartite ATP-independent periplasmic TRAP transporter
Source: Nat Commun. 2022 Aug 4;13:4471. doi: 10.1038/s41467-022-31907-y (PMC9352664; doi:10.1038/s41467-022-31907-y)
Supplement: Supplementary file 1 — Supplementary Information [file 41467_2022_31907_MOESM1_ESM.pdf]

Supplementary Information for:

Structural and mechanistic analysis of a tripartite ATP-independent periplasmic TRAP transporter

Martin F. Peter[1], Jan A. Ruland[2], Peer Depping[1,3], Niels Schneberger[1], Emmanuele Severi[4,5], Jonas Moecking[1], Karl Gatterdam[1], Sarah Tindall[4], Alexandre Durand[6], Veronika Heinz[7], Jan-Peter Siebrasse[2], Paul-Albert Koenig[8], Matthias Geyer[1], Christine Ziegler[7], Ulrich Kubitscheck[2], Gavin H. Thomas[4], Gregor Hagelueken[1,*]

[1] Institute of Structural Biology, University of Bonn, Venusberg-Campus 1, 53127 Bonn, Germany

[2] Institute for Physical und Theoretical Chemistry, University of Bonn, Wegelerstr. 12, 53127 Bonn, Germany

[3] Aston Centre for Membrane Proteins and Lipids Research, Aston St., B4 7ET, Birmingham, United Kingdom

[4] Department of Biology (Area 10), University of York, York YO10 5YW, United Kingdom

[5] Biosciences Institute, Newcastle University, Newcastle, NE2 4HH, United Kingdom

[6] Institut de Génétique et de Biologie Molecule et Cellulaire, 1 Rue Laurent Fries, 67404 Illkirch CEDEX, France

[7] Institute of Biophysics and Biophysical Chemistry, University of Regensburg, 95053 Regensburg, Germany

[8] Core Facility Nanobodies, University of Bonn, Venusberg-Campus 1, 53127 Bonn, Germany

*Corresponding author email: hagelueken@uni-bonn.de

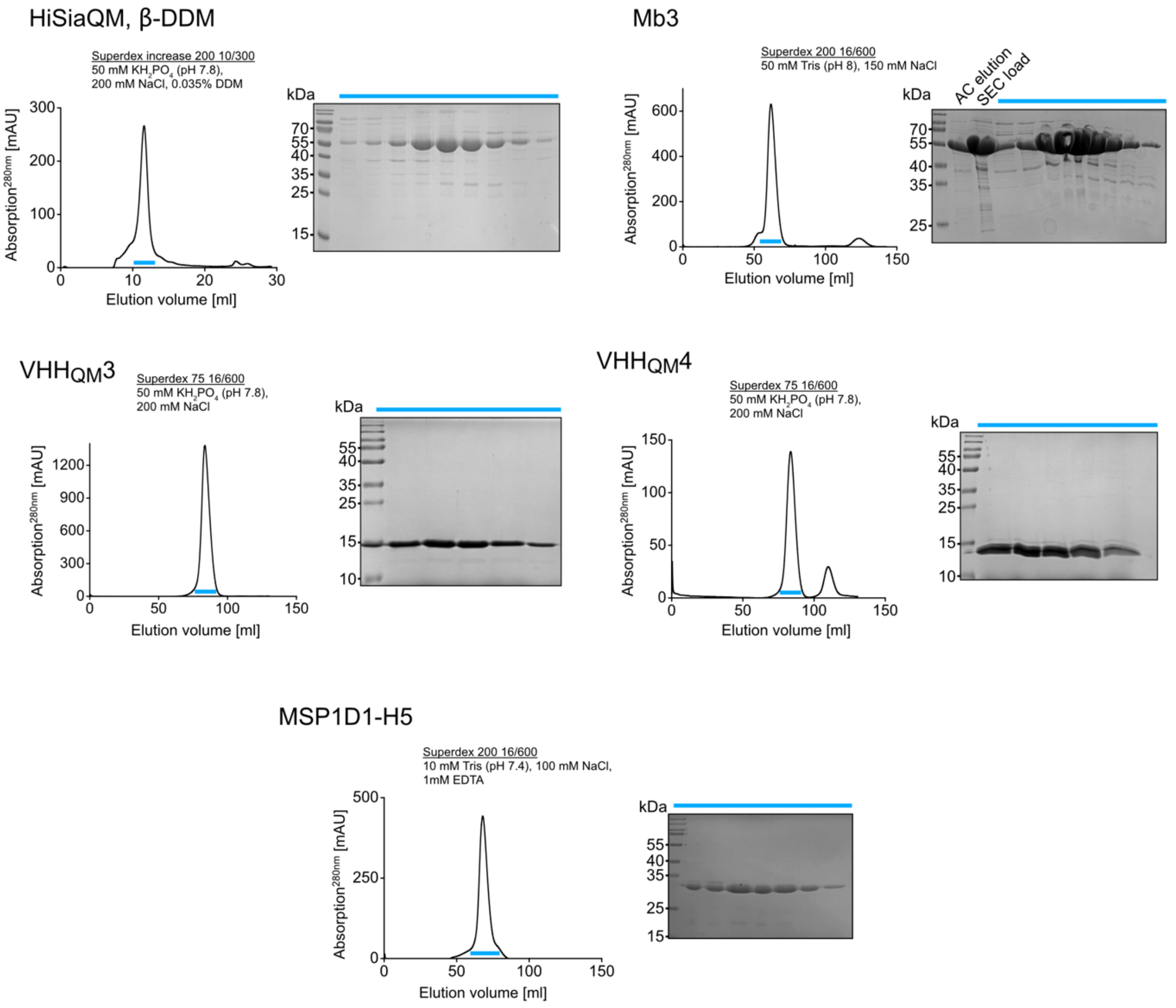

Supplementary Fig. 1 | Quality of proteins used in this study. A size-exclusion chromatography profile is shown for each protein. The buffers and SEC columns are given next to the chromatograms. The fractions highlighted in blue were analysed by SDS-PAGE, as shown on the right side of the corresponding SEC profile. The panels show representative examples of n ≥3 experiments. Source data are provided as a Source Data file.

a

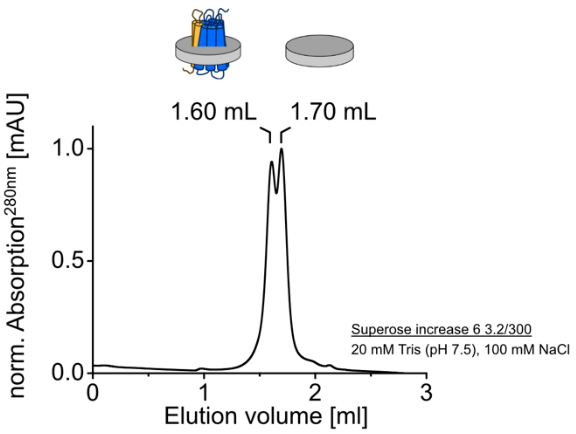

b

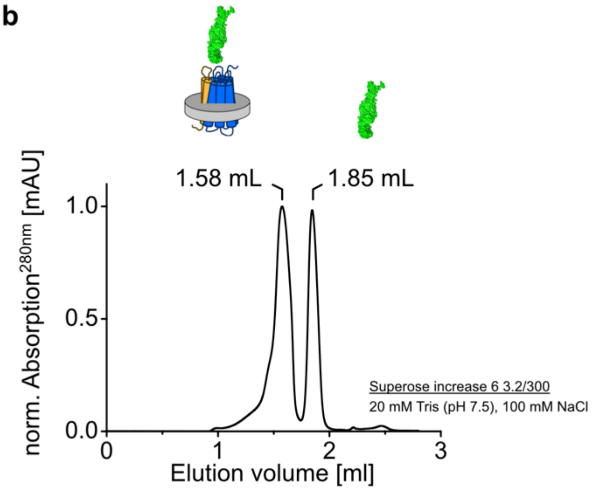

c

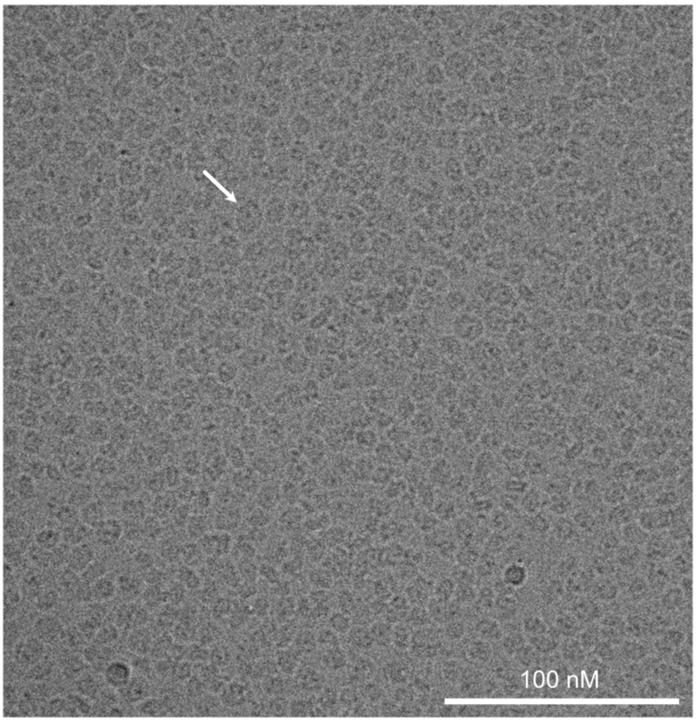

Supplementary Fig. 2 | Cryo-EM sample preparation and raw micrograph of the HiSiaQM/Mb3 complex in MSP1D1-H5 nanodiscs. a, Normalized SEC elution profile of the HiSiaQM-MSP1D1-H5 reconstitution mixture. **b,** Normalized SEC elution profile of the HiSiaQM-MSP1D1-H5 reconstitution mixture after Ni-NTA affinity chromatography pull-down via immobilized Mb3. **c,** The picture was recorded with a ThermoFisher Glacios microscope equipped with a K2 camera at 200 kV. The white arrow marks a representative nanodisc in top-view with Mb3 visible as a dark spot. The micrograph is a representative example of the n=5004 micrographs recorded of the sample.

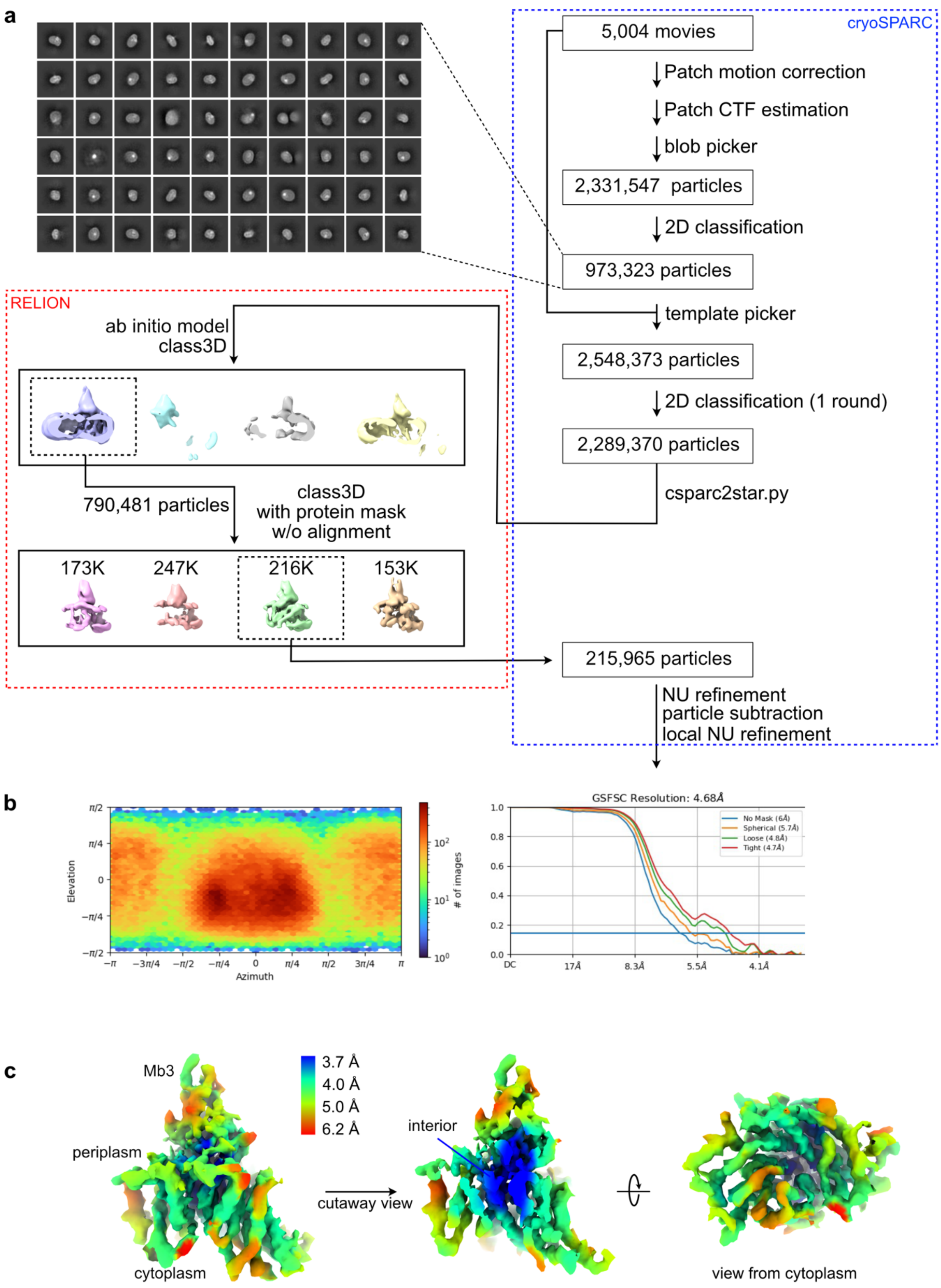

Supplementary Fig. 3 | Cryo-EM processing workflow. a, Workflow for the 3D reconstruction of HiSiaQM in lipid nanodiscs with Mb3 bound to the periplasmic side. Dashed blue and red boxes indicate which parts of the process were performed in cryoSPARC [1] or RELION [2]. **b,** Distribution of viewing angles and Gold-standard FSC curves for the final refinement step. **c,** Local resolution estimates calculated in cryoSPARC (0.143 cutoff).

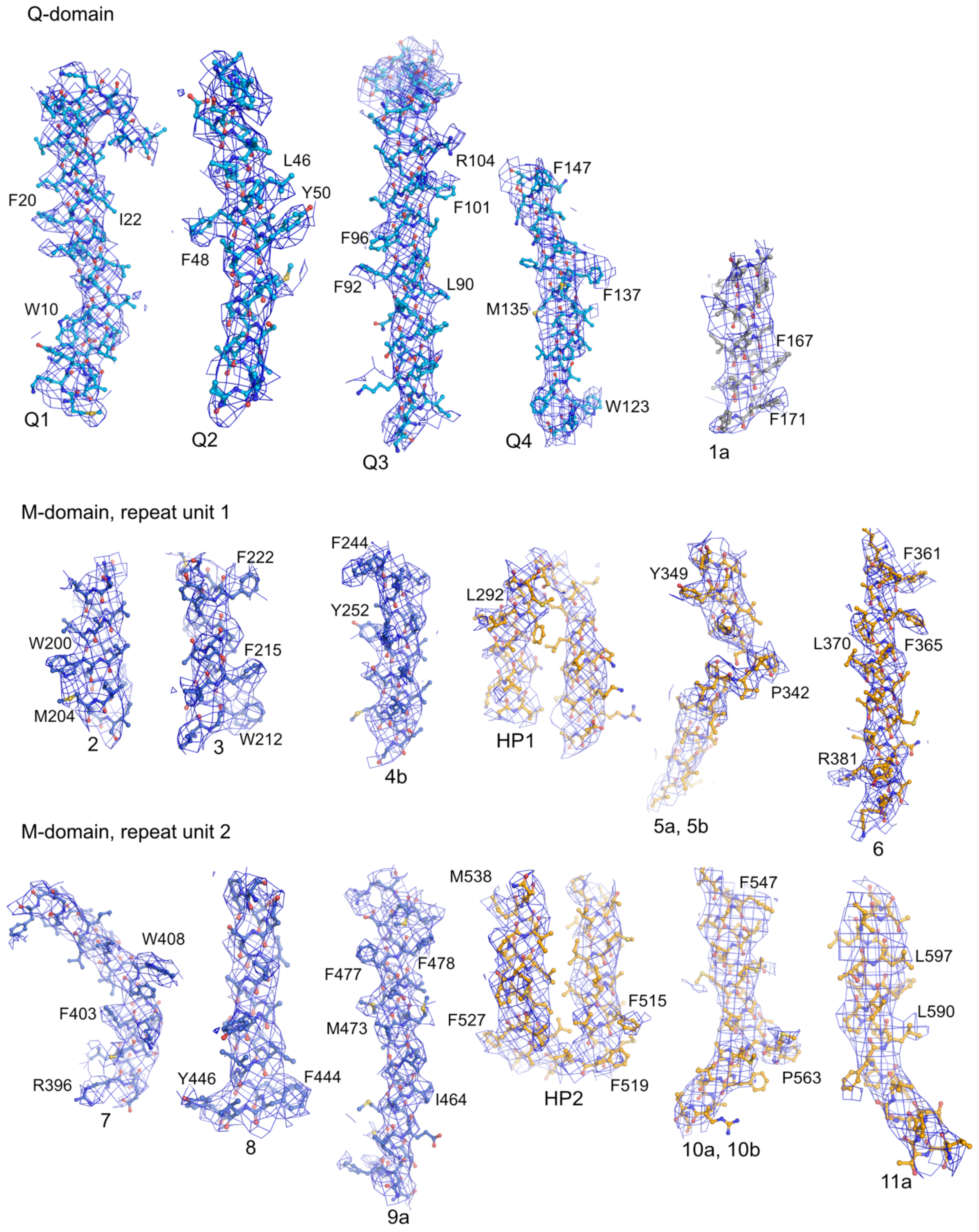

Supplementary Fig. 4 | Fit of TM helices of HiSiaQM to the 3D reconstruction. The transmembrane helices (number is given at the bottom of each helix, compare Figure 1) are shown as ball-and-stick models. The color code is defined in Figure 1. The blue mesh is the 3D reconstruction at a sigma level of 6.0. Selected residues are indicated.

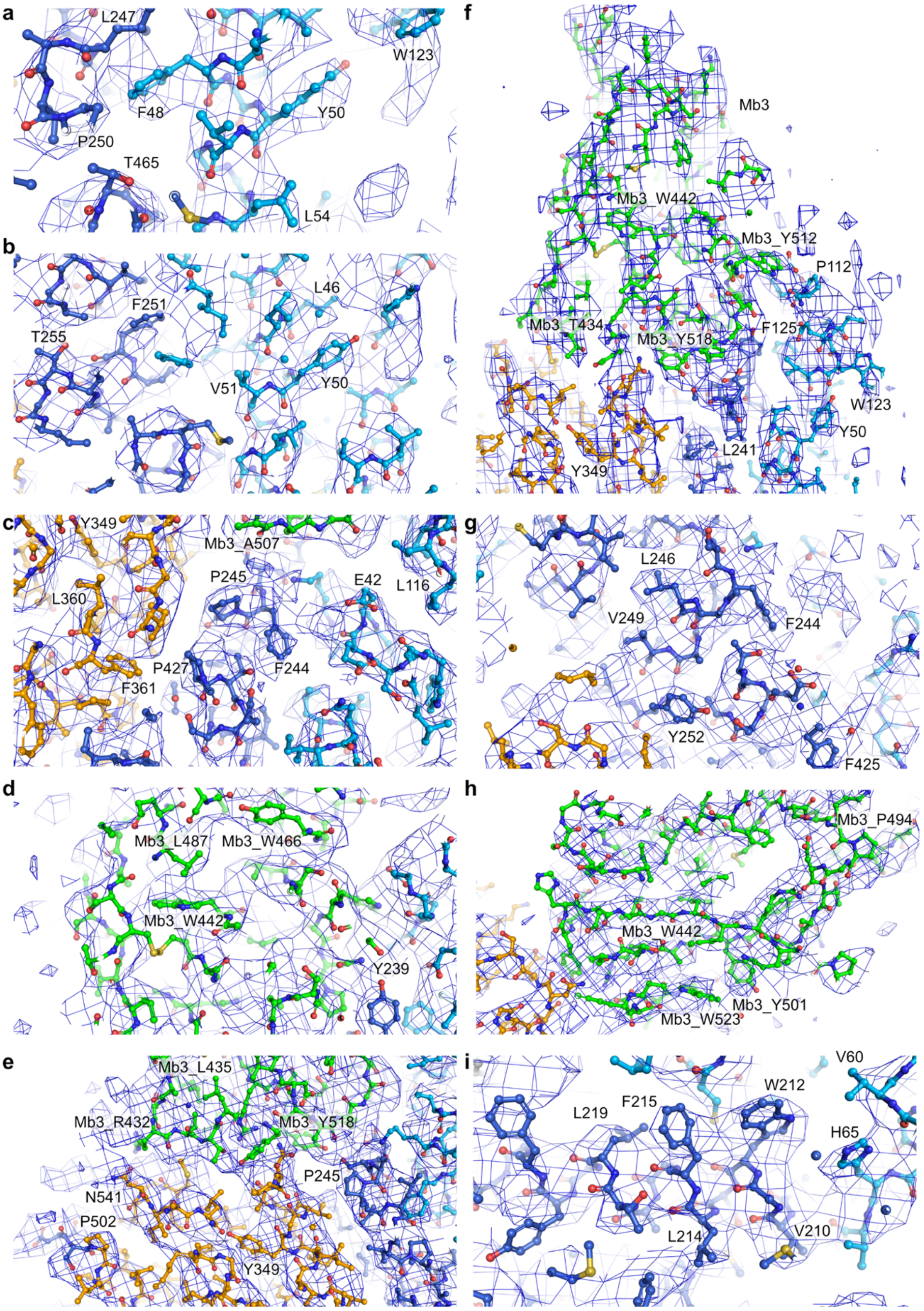

Supplementary Fig. 5 | Fit of the HiSiaQM model to the 3D reconstruction. The HiSiaQM model is shown in ball-and-stick representation. The color code is defined in Figure 1. The blue mesh is the 3D reconstruction at a sigma level of 6.0. Selected residues are indicated. **a)** Detail of the QM interface. **b)** Detail of the QM interface. **c)** Detail of the stator/elevator/VHH$_{QM}$3 interface. **d)** Core region of VHH$_{QM}$3. **e, f)** Detail of the stator/elevator/VHH$_{QM}$3 interface. **g)** Detail of the stator/elevator interface. **h)** Detail of VHH$_{QM}$3. **i)** Detail of the QM interface.

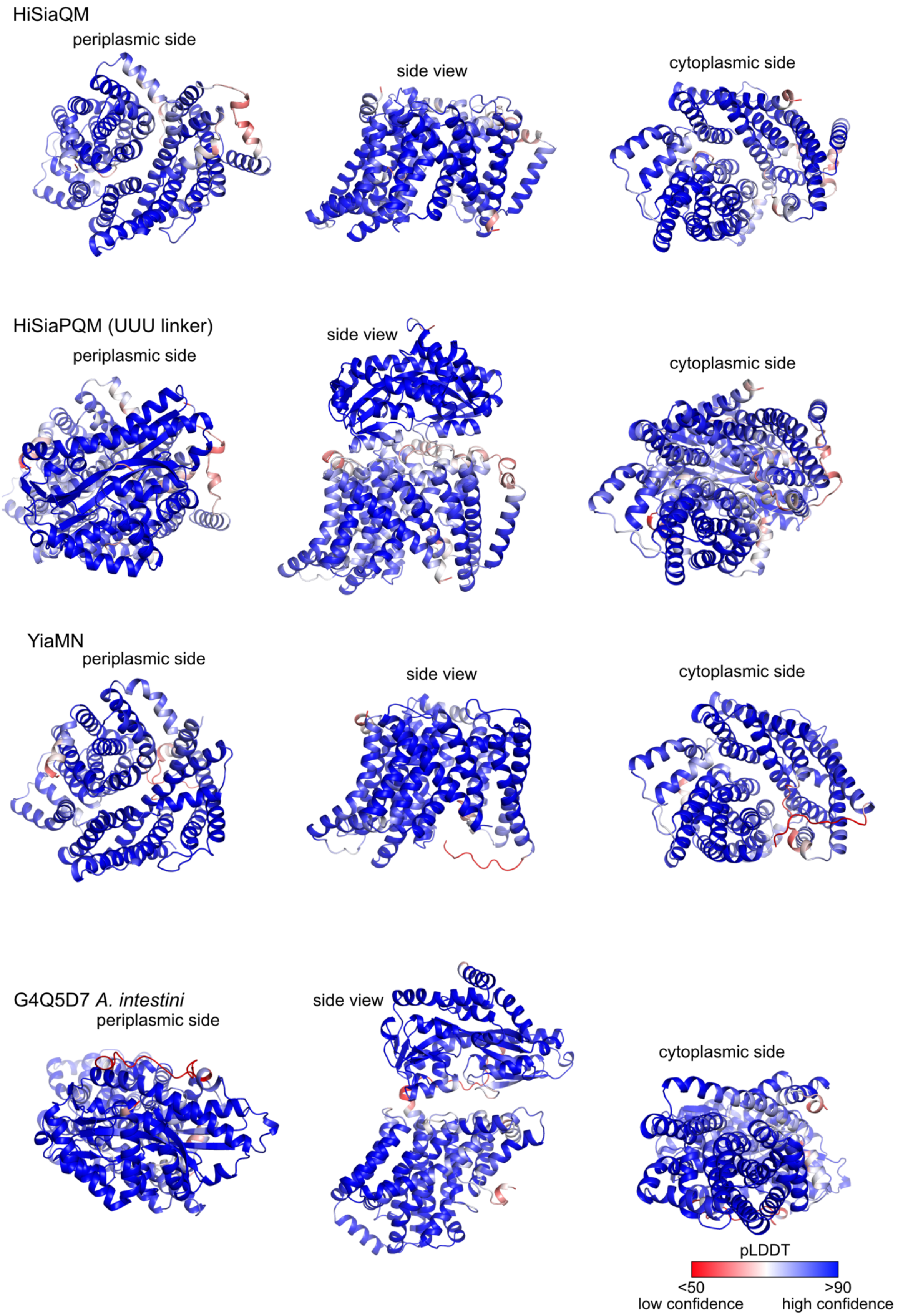

Supplementary Fig. 6 | AlphaFold models that were used in this study. The pLDDT confidence score is indicated by a color gradient.

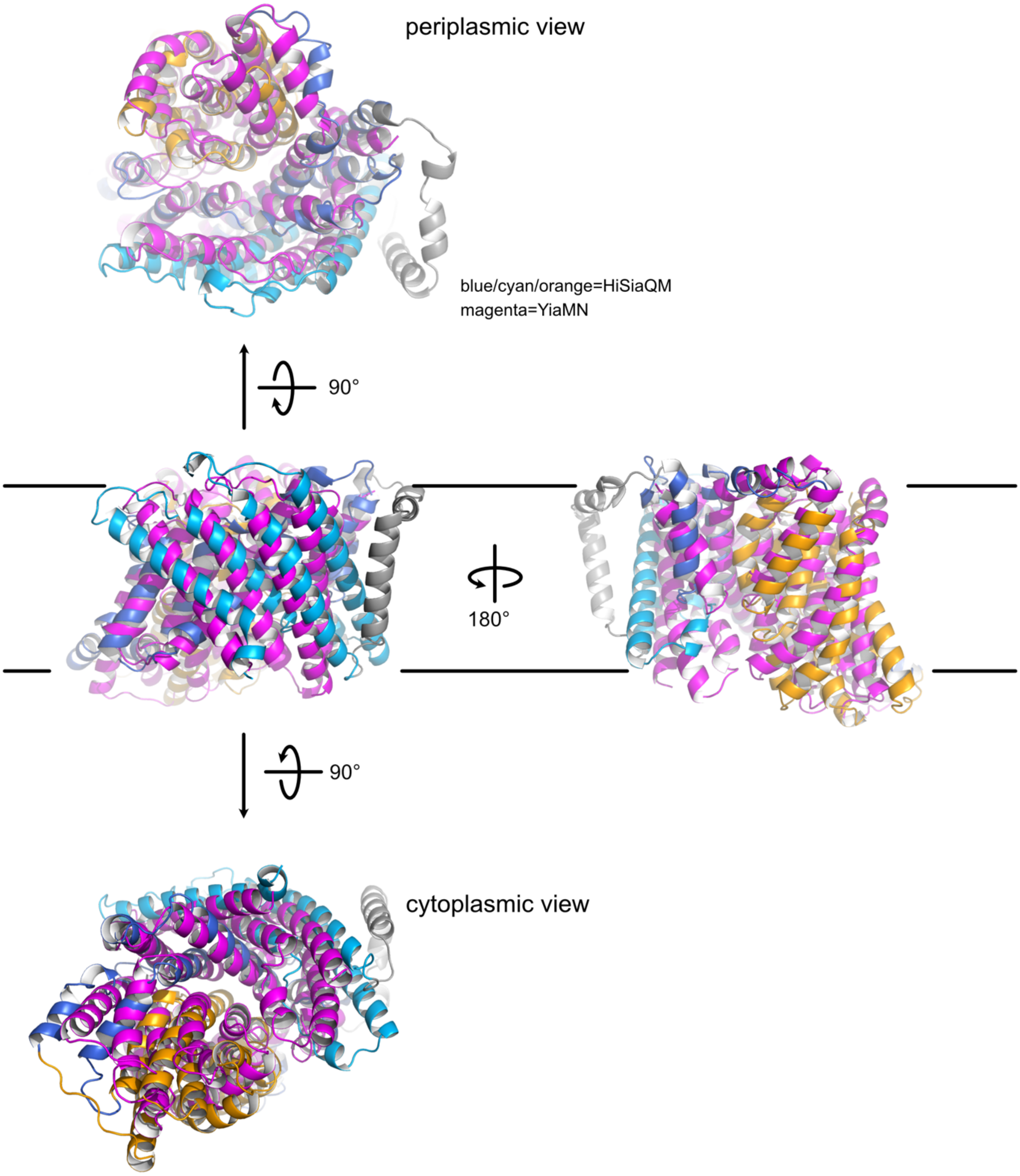

Supplementary Fig. 7 | Comparison of the predicted structure of YiaMN to the experimental HiSiaQM structure. The YiaMN model (magenta) is from Ovchinnikov et al. [3]. The HiSiaQM model was determined in this study with the color code as defined in Figure 1.

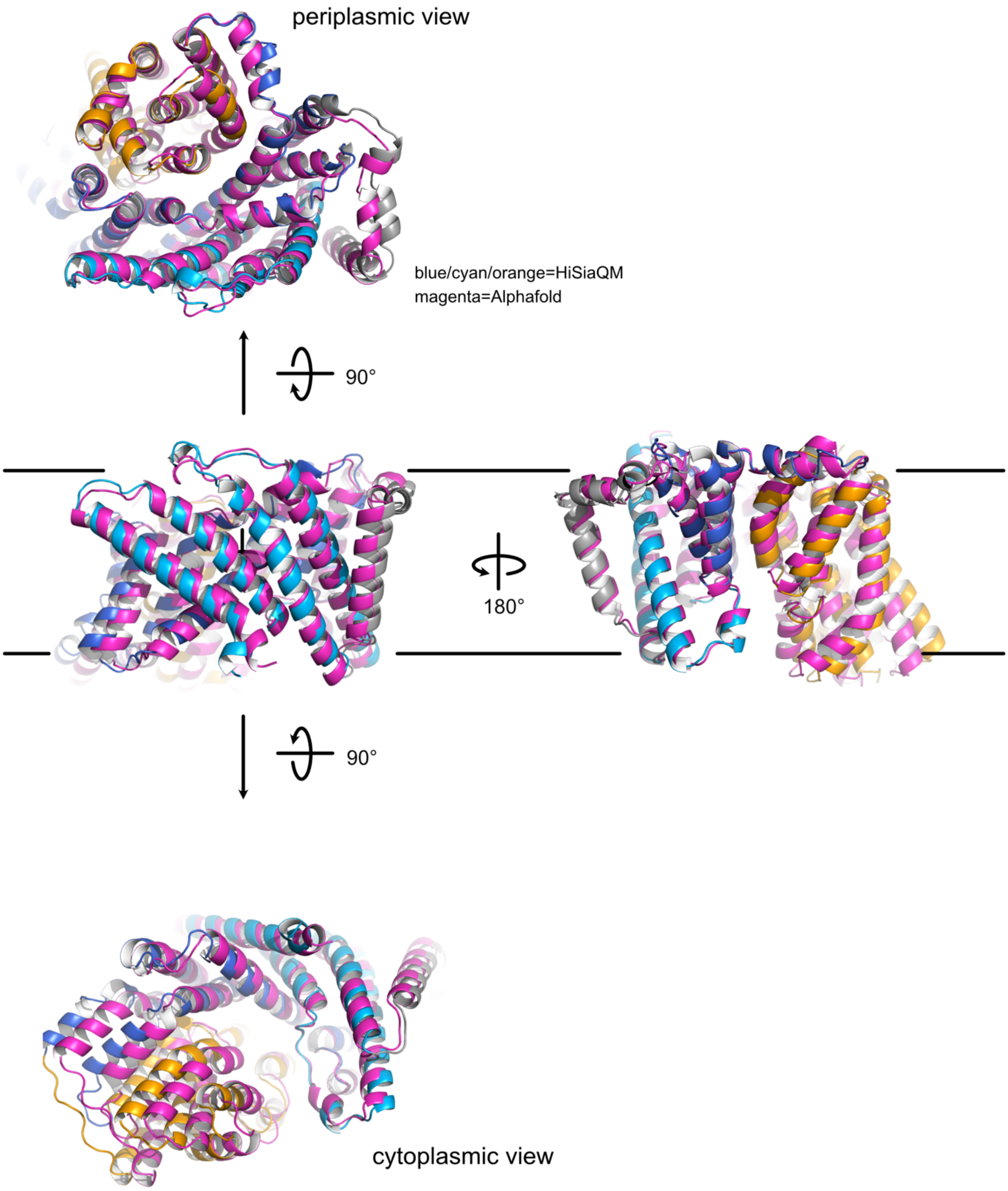

Supplementary Fig. 8 | Alignment of the in lipid experimental HiSiaQM structure to the AlphaFold model. The experimental structure is shown with the same color code as defined in Figure 1. The Alphafold[4] model is colored magenta.

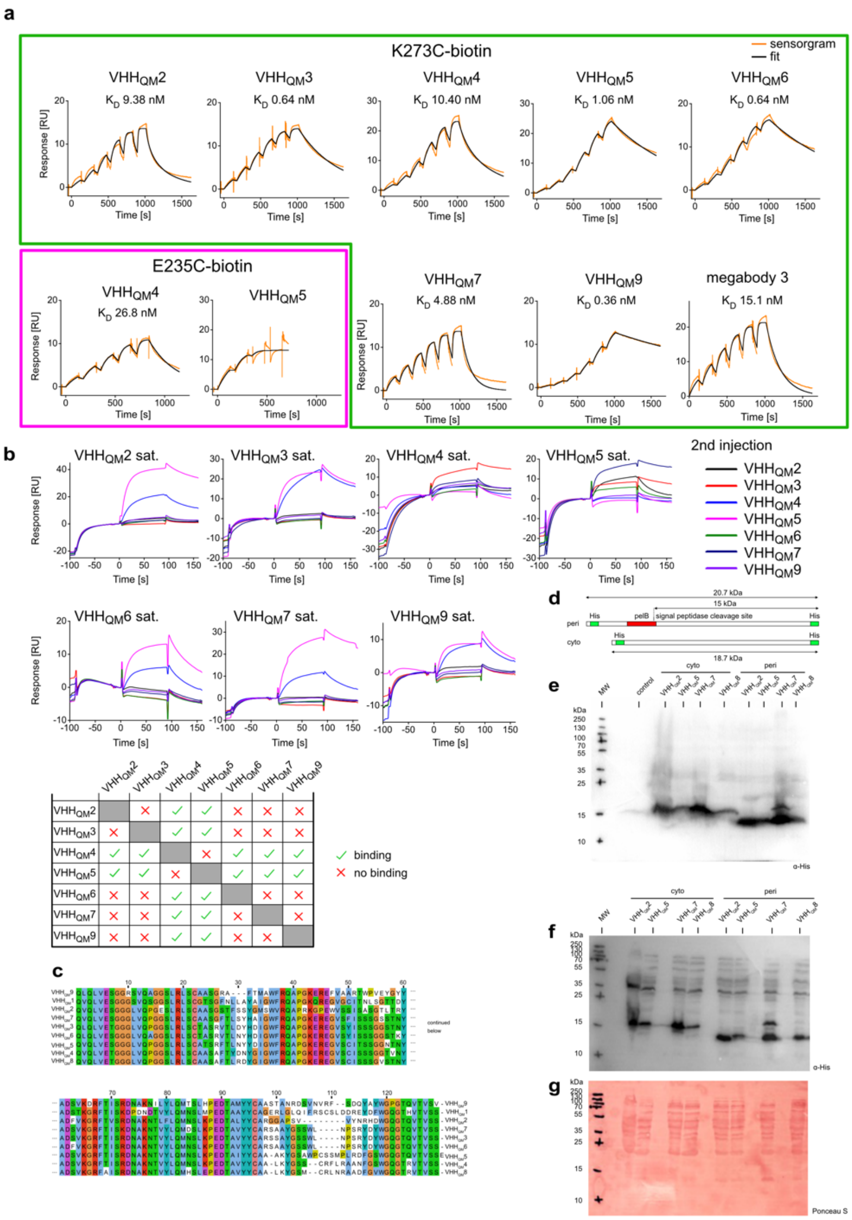

Supplementary Fig. 9 | SPR data on HiSiaQM specific VHHs. a, single cycle kinetics of the different VHHs**.** The green and magenta boxes group experiments with two different positions of the biotin label that was used for immobilization. **b,** HiSiaQM/VHH interaction was analyzed in a competitive binding experiment in which the TRAP transporter (HiSiaQM K273C-biotin), immobilized on an SPR chip, was saturated with a first VHH and the binding behavior of a second VHH was observed. The primary VHH is indicated next to the chromatogram and the secondary VHH is given by the color code. Bottom: Matrix summarizing the epitope binding results. **c,** Amino acid sequences of the VHH$_{QM}$s used in this study. **d**, Constructs used to test the effect of different VHHs on Neu5Ac uptake by HiSiaPQM. The position of the pelB signal sequence and the signal peptidase cleavage site, as well as the location of the His tags are marked. **e**, Western blot (anti His) showing the expression levels of VHH$_{QM}$2,5,8 (weak or no inhibition of uptake) relative to VHH$_{QM}$7 (strong inhibition of uptake). Note that the SPR data represent a single set of experiments. The blot was independently repeated twice. **f**, The VHH samples from e) with one fourth of the amount of sample loaded to allow identification of single bands. **g,** Ponceau S stain of the blot in f) as loading control. Source data are provided as a Source Data file.

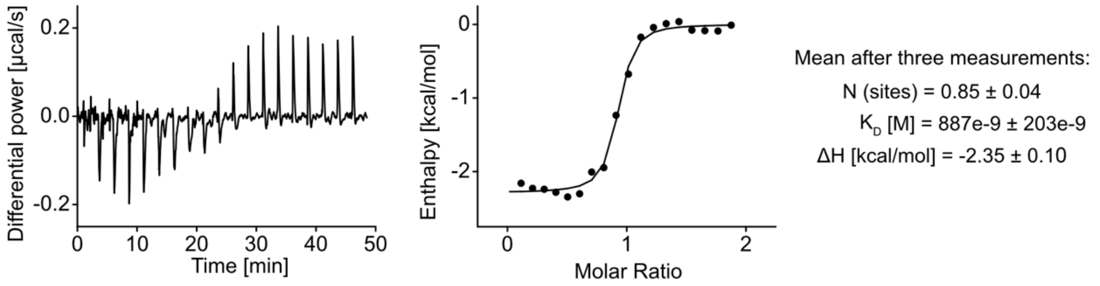

Supplementary Fig. 10 | Characterization of HiSiaP/VHH$_P$1 binding via ITC. Isothermal titration calorimetry experiment between HiSiaP and the HiSiaP-VHH (left) and binding curve (middle). The thermodynamic parameters from the experiment are shown on the right and are derived from n=3 independent experiments. Source data are provided as source data file.

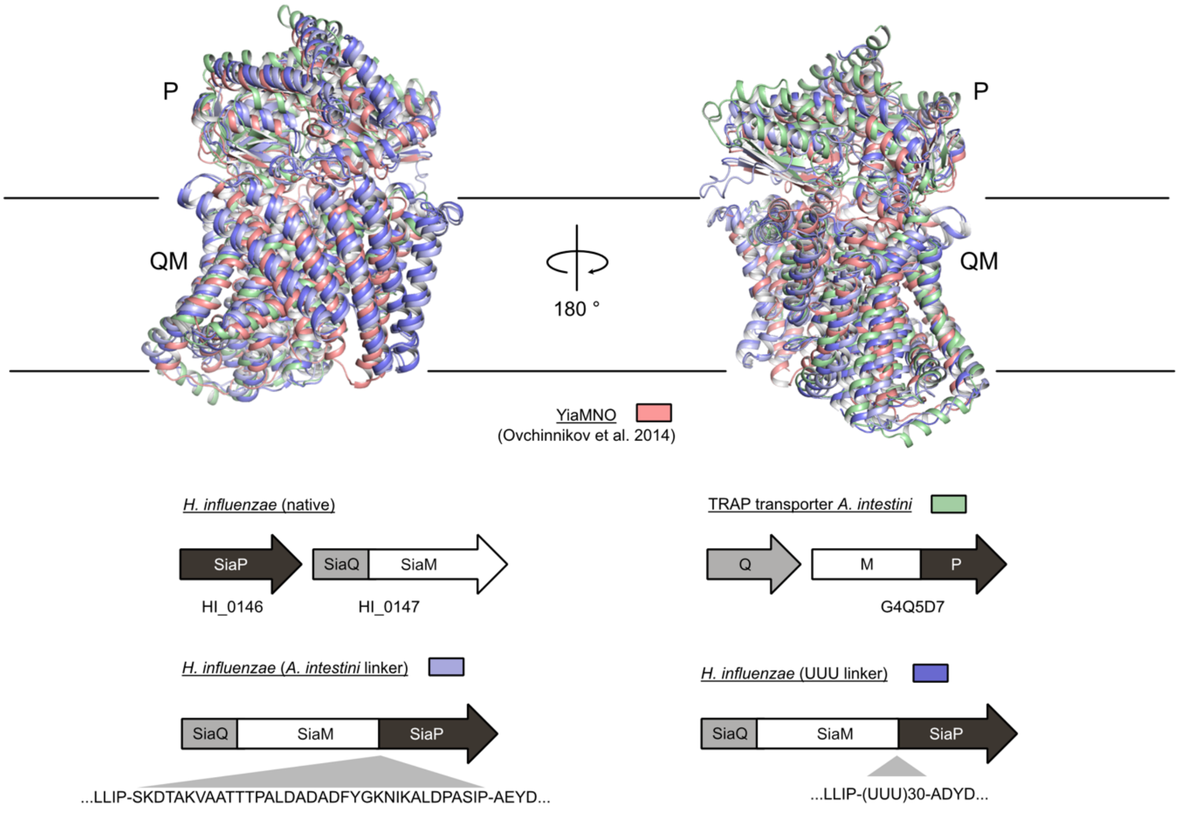

Supplementary Fig. 11 | Comparison of models for the tripartite complex. Four different models of the tripartite complex aligned onto each other. The colors are indicated below, next to the organization of the corresponding genes. If domains were fused for modelling, the linkers are specified next to genes. Confidence scores for the different AlphaFold models are shown in Supplementary Fig. 6.

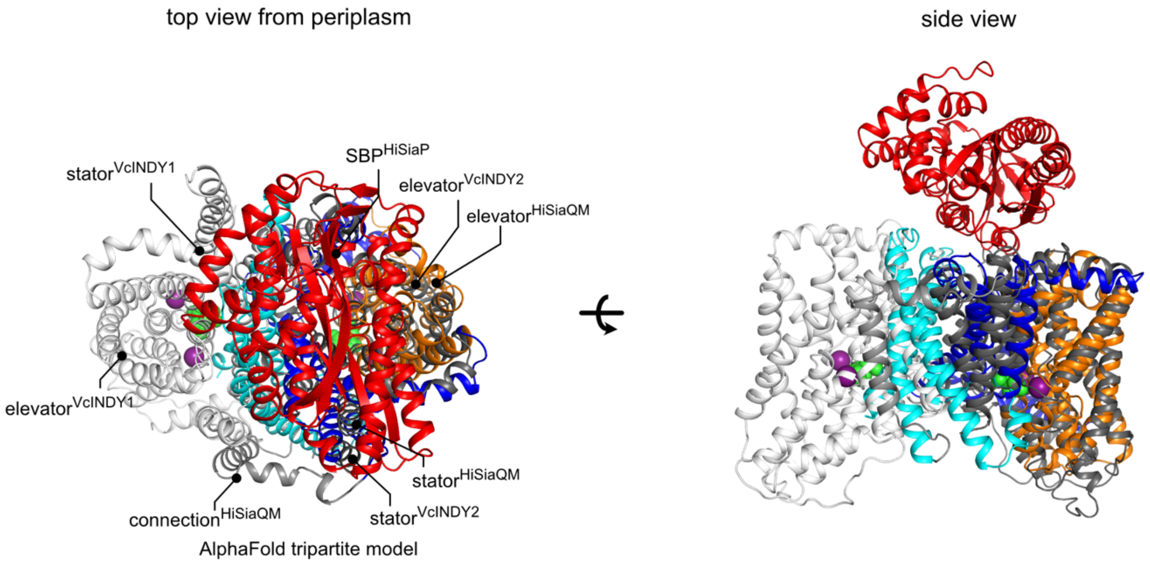

Supplementary Fig. 12 | Alignment of the TRAP transporter HiSiaPQM with VcINDY. Alignment of the tripartite HiSiaPQM AlphaFold model (colored as in Figure 1) from Figure 4 to VcINDY (white, 5UL9 [http://doi.org/10.2210/pdb5UL9/pdb]). The HiSiaP SBP is shown in red.

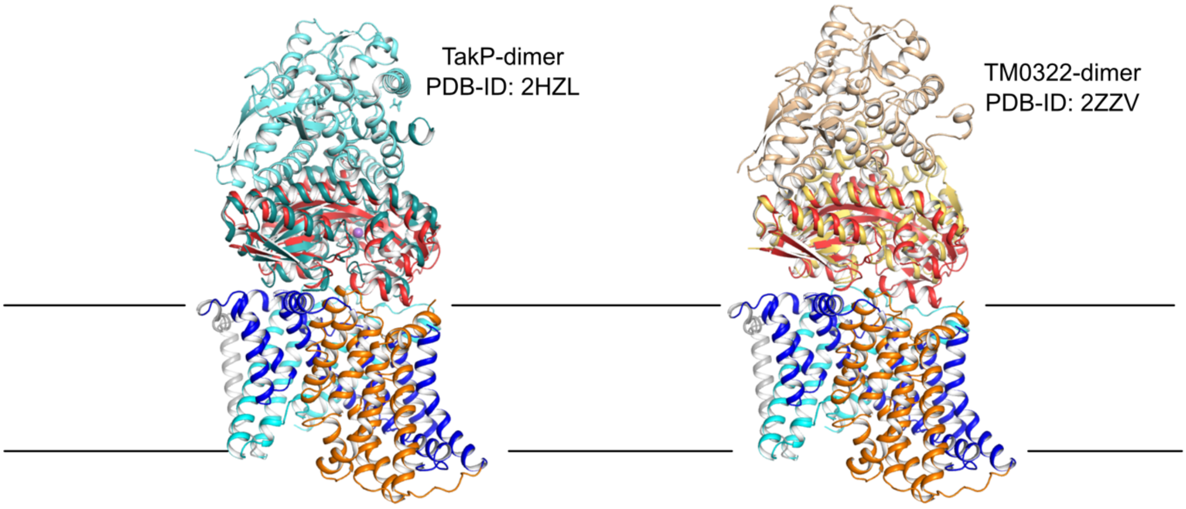

Supplementary Fig. 13 | Orientation of dimeric SBPs in the model of the tripartite complex. One monomer of the two different dimeric SBPs, TakP (green-blue; 2HZL [http://doi.org/10.2210/pdb2HZL/pdb]) and TM0322 (yellow-wheat; 2ZZV [http://doi.org/10.2210/pdb2ZZV/pdb]), was aligned on the HiSiaP SBP (red) in the tripartite complex as predicted by AlphaFold (Figure 4).

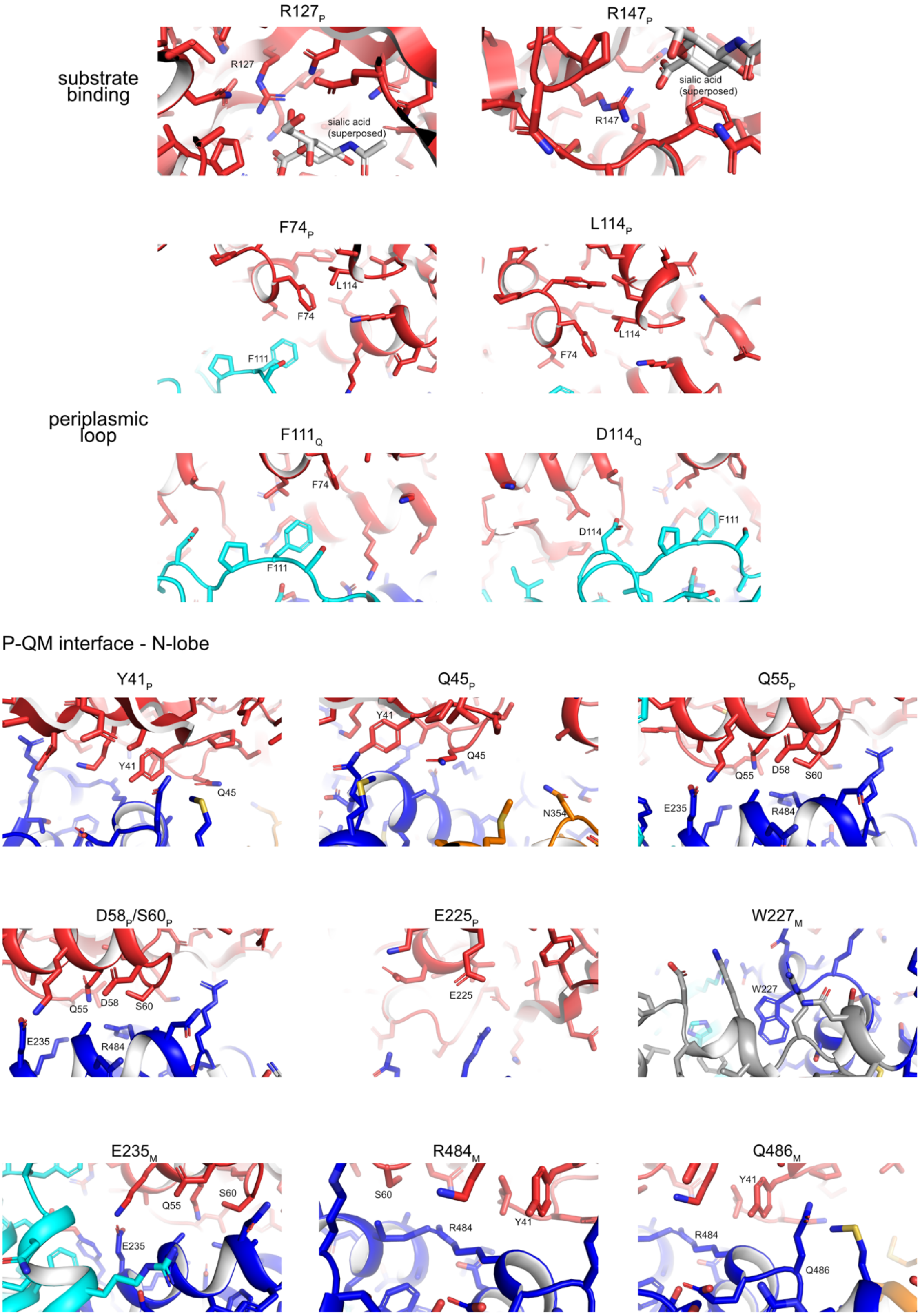

(Figure is continued on the next page)

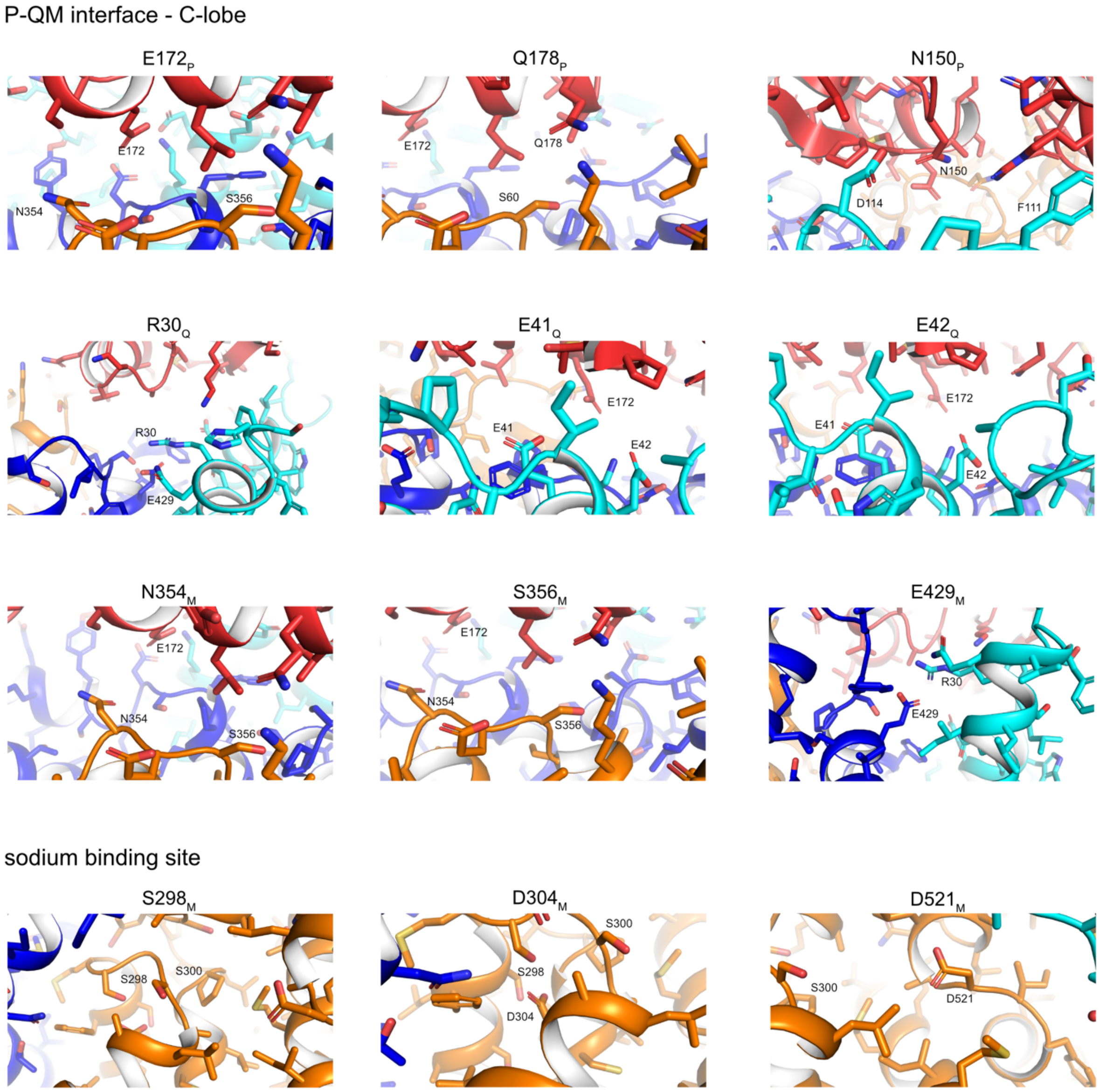

Supplementary Fig. 14 | Close-up views of mutants from Figure 5. All residues that were analyzed in the complementation assay (Figure 5) in the main text are shown in detail in the AlphaFold model of the tripartite complex.

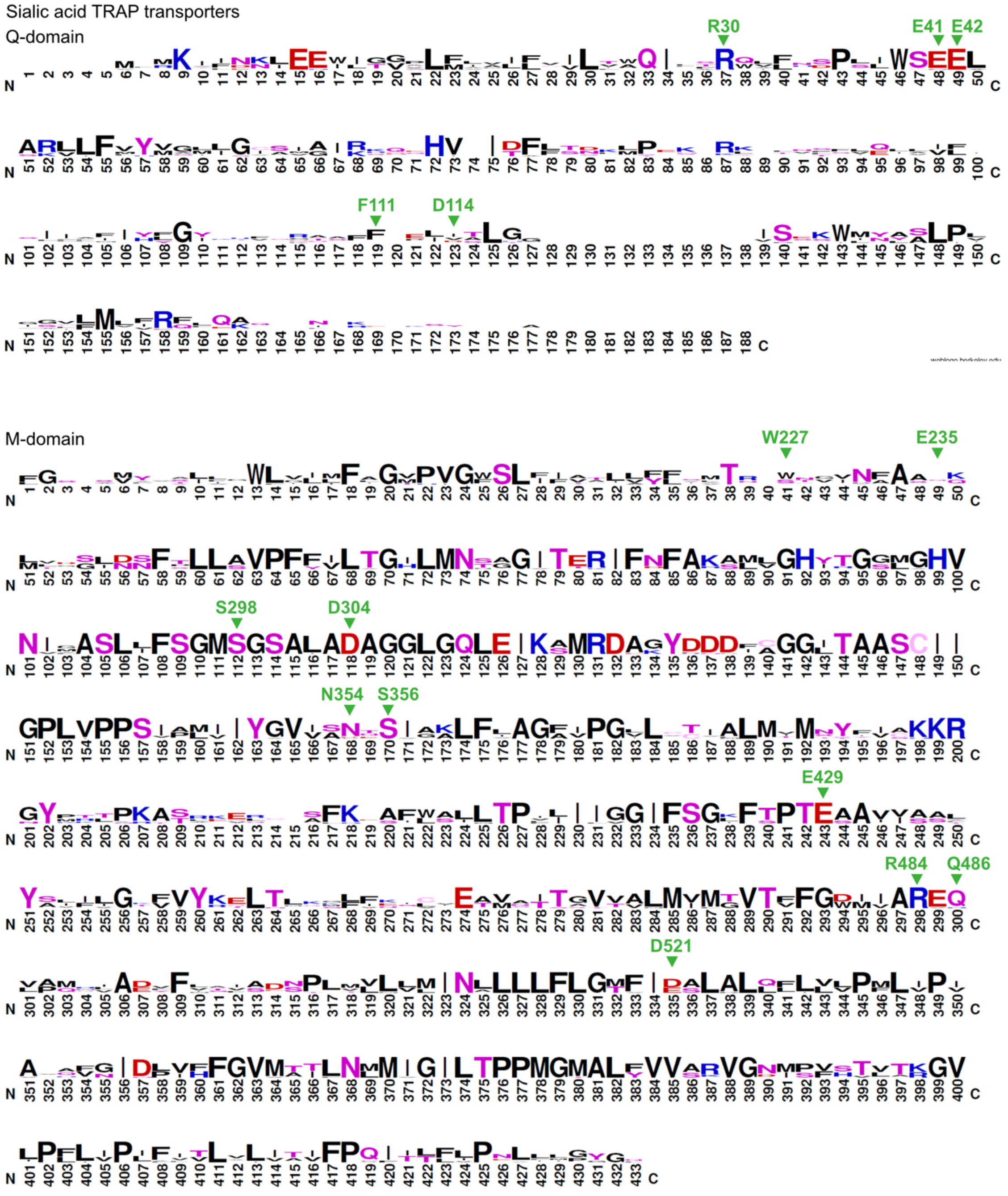

Supplementary Fig. 15 | Sequence conservation of sialic acid TRAP transporters. Positions in HiSiaQM that were mutated and analyzed in the complementation assay in Figure 5 are marked with green triangles with the HiSiaQM numbering indicated. Sequences (see below) were taken from Chowdhury et al. [5] and Vetting et al. [6], with just using those with known ligands. Here, just sialic acid bound TRAP transporters are shown, the conservation of TRAP transporters which do not transport sialic acid is shown in Supplementary Fig. 16. The QM sequences were split into Q and M for the alignment using HiSiaQM as a guide. The sequences were aligned using ClustalO[7] in Jalview[8] with default settings and sequence logo motifs generated using WebLogo [9]. The underlying sequences are specified in Supplementary Table 5.

TRAP transporters
Q-domain

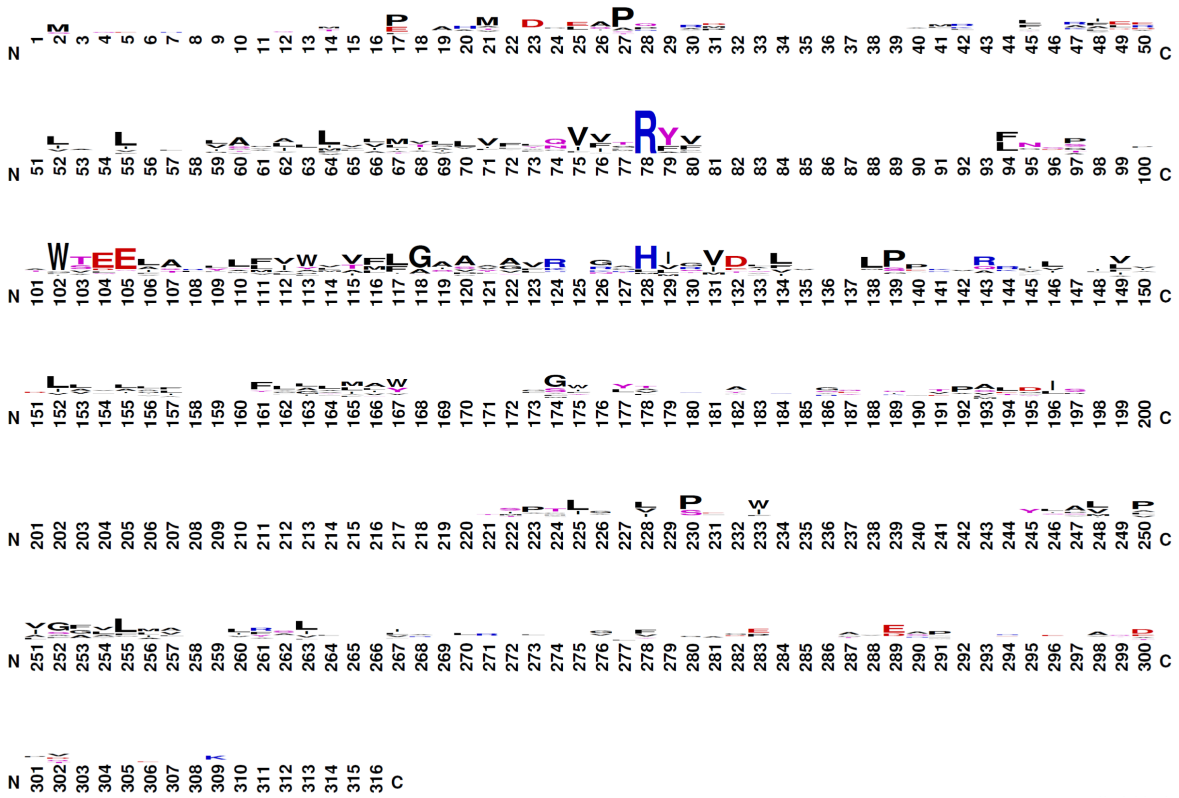

(Figure is continued on the next page)

TRAP transporters

M-domain

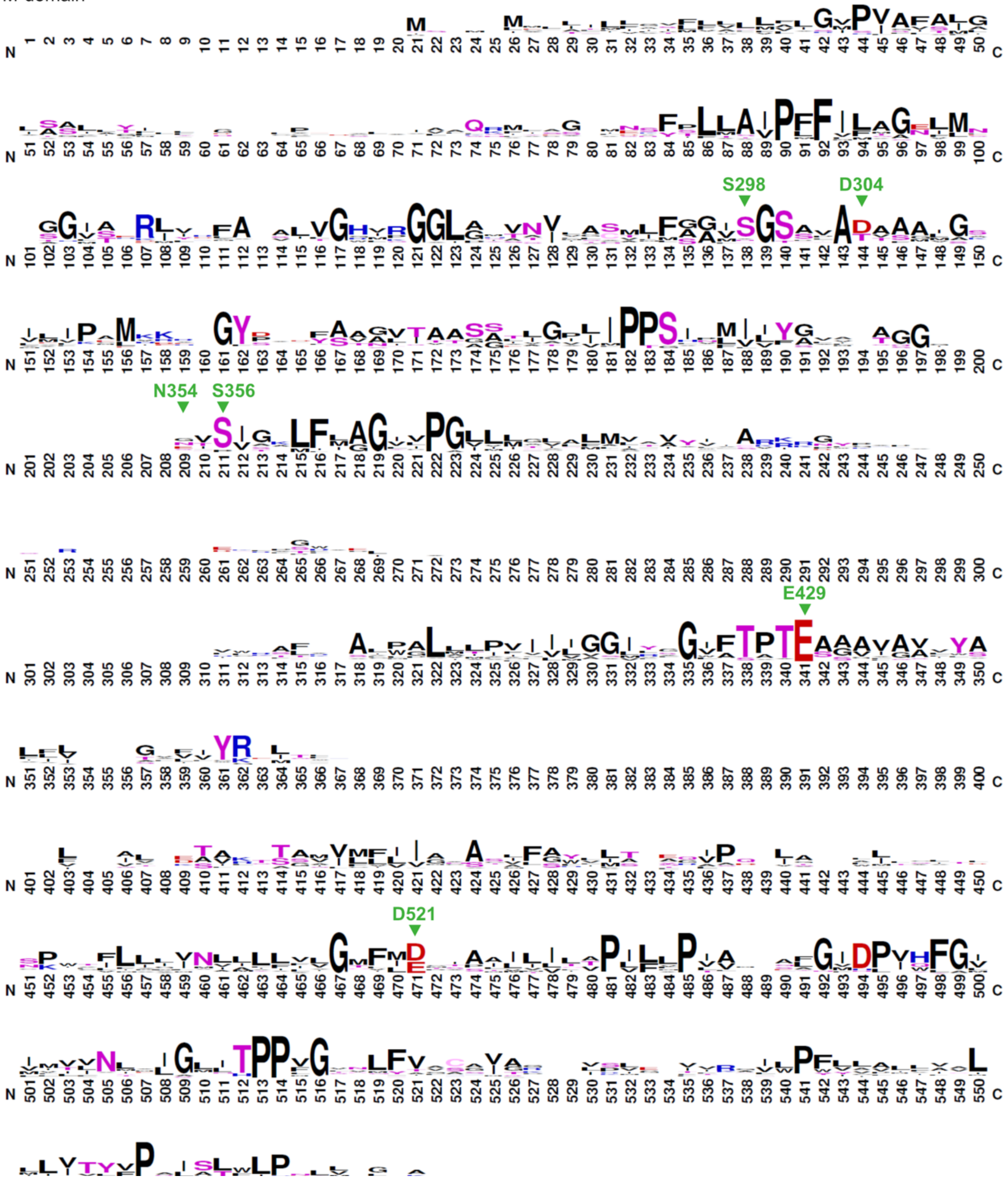

Supplementary Fig. 16 | Sequence conservation of TRAP transporters. Positions in HiSiaQM that were mutated and analyzed in the complementation assay in Figure 5 are marked by green triangles with the HiSiaQM numbering. As for Figure S15, sequences (see below) were taken from Chowdhury et al. [5] and Vetting et al. [6], with just using those with known ligands. In contrast to Supplementary Fig. 15, this alignment shows TRAP transporters which do not transport sialic acid. The QM sequences were split into Q and M for alignment using HiSiaQM as a guide. The sequences were aligned using ClustalO [7] in Jalview [8] with default settings and sequence logo motifs generated using WebLogo [10]. The underlying sequences are specified in Supplementary Table 6.

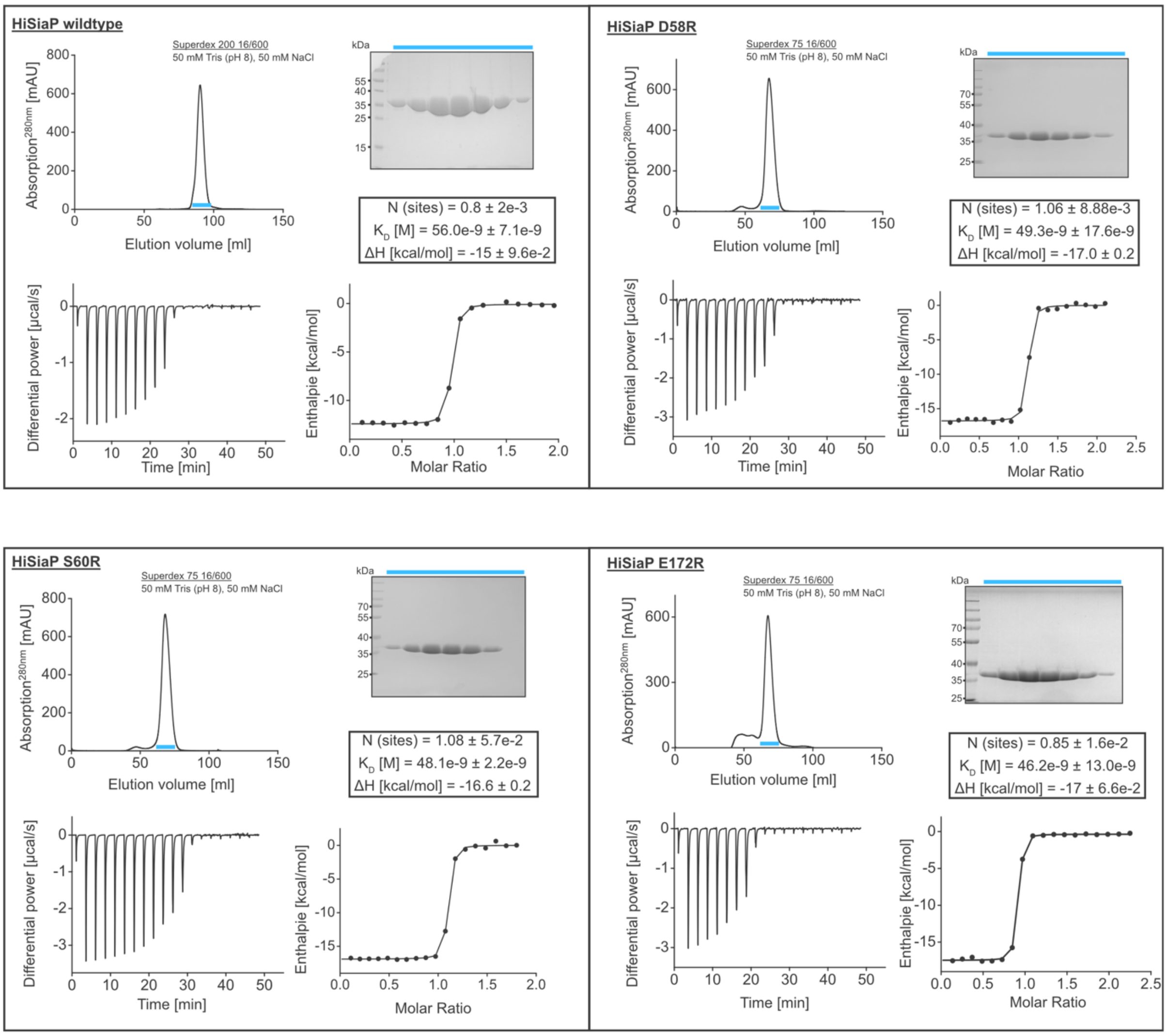

Supplementary Fig. 17 | Gelfiltration and isothermal titration calorimetry analysis of P domain mutants. The binding parameters were determined from n=3 independent experiments. Source data are provided as source data file.

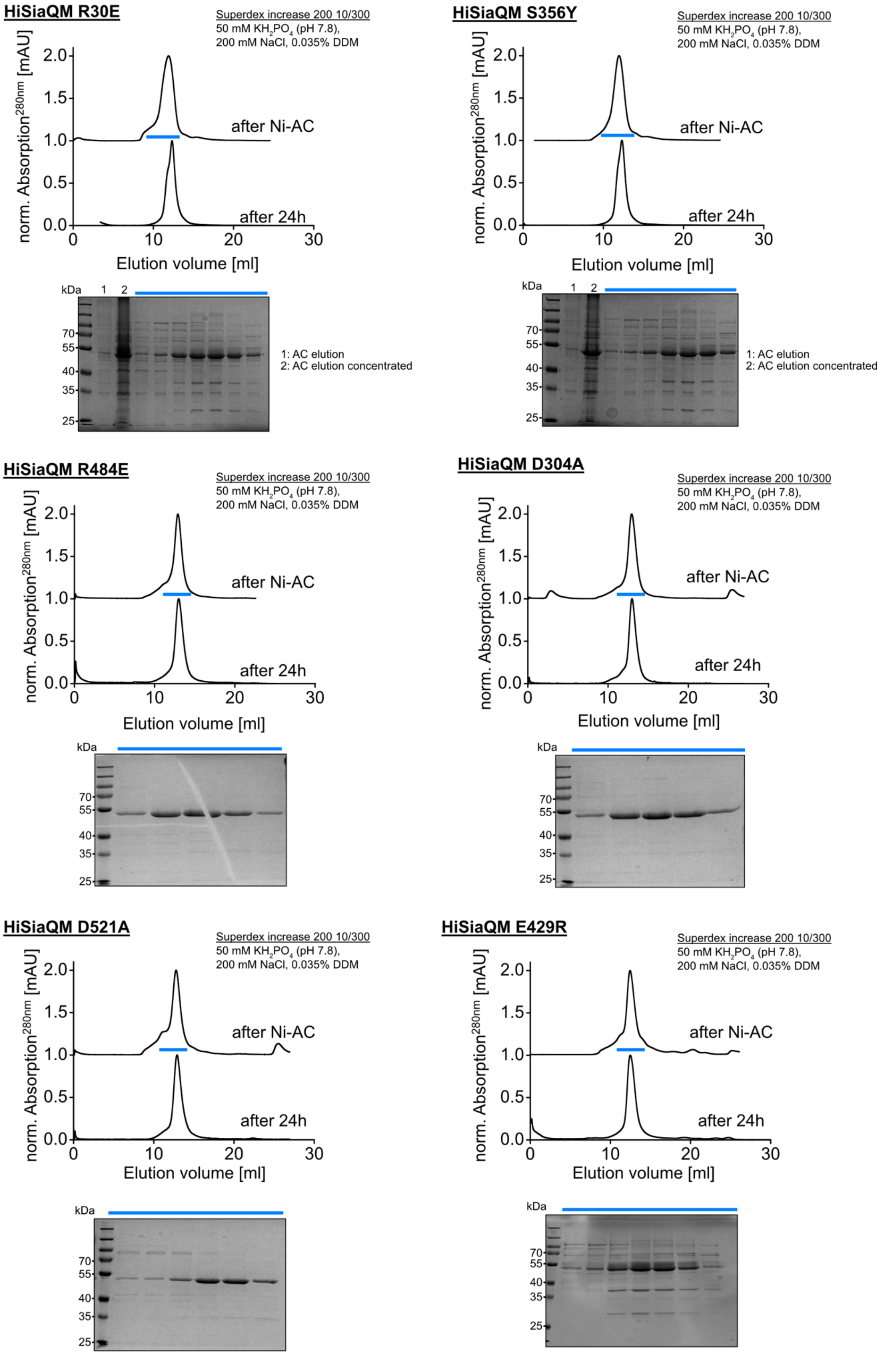

Supplementary Fig. 18 | Expression and gelfiltration analysis of QM-domain mutants. The mutants were expressed and purified once.

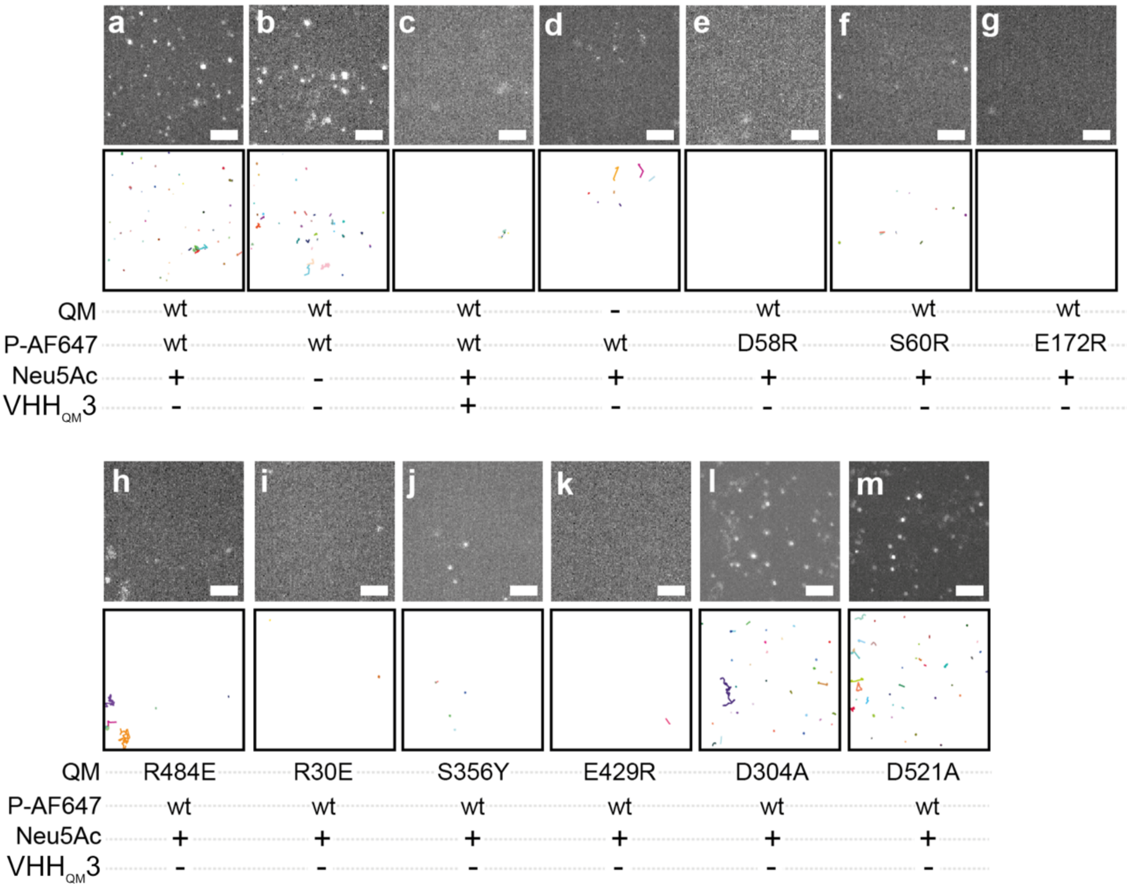

Supplementary Fig. 19 | a-m, Maximum intensity projections (upper row) and raw traces (bottom row) of the measurements shown in Figure 6. All experiments were independently performed three or more times (n ≥3). The scale bars equal 3 µm.

Supplementary Table 1 | Cryo-EM data collection and processing

**Sample**	HiSiaQM/Mb3 in MSP1D1-H5 nanodiscs
**Data acquisition**	
Voltage	300 kV
Magnification	81,000
Pixel size	0.862 Å
Electron dose	50.213 $e^{-}/Å^{2}$
Frame number	50
Defocus range	-0.8 to -2.5 µm
**Data processing & Refinement**	
EMDB code	EMD-13930
PDB code	7QE5
Symmetry imposed	No symmetry imposed
Initial number of particles	2,331,547
Final number of particles	215,956
Map resolution at 0.143 FSC	4.7 Å
Local resolution range	3.7-6.2 Å
Map sharpening B-factor	-256 $Å^{2}$
**Refinement**	
Initial PDBs used	AlphaFold model of HiSiaQM
Non hydrogen atoms	5,720
Protein	5,720
Ligands	n/a
**Mean B factors ($Å^{2}$)**	
Protein	105.81
Ligand	n/a
**R. m. s. deviations**	
Bond lengths (Å)	0.013
Bond angles (°)	2.056
**Model validation**	
MolProbity [11] score	1.83
Clash score	8.07
Rotamer outliers (%)	1.14
C-beta outliers (%)	2.5
**Ramachandran plot**	
Favored (%)	94.96
Allowed (%)	4.77
Outliers (%)	0.27

Supplementary Table 2 | SPR binding parameters of VHHs and megabody on K273C-biotin (top) and E235C-biotin (bottom).

	K_D [nM]	k_{off} [$M^{-1}s^{-1}$]	k_{on} [s^{-1}]
**VHH$_{QM}$2**	9.83	$1.94*10^6$	$19.0*10^{-3}$
**VHH$_{QM}$3**	0.64	$3.01*10^6$	$1.91*10^{-3}$
**VHH$_{QM}$4**	10.40	$0.47*10^6$	$4.92*10^{-3}$
**VHH$_{QM}$5**	1.06	$1.01*10^6$	$1.07*10^{-3}$
**VHH$_{QM}$6**	0.64	$1.54*10^6$	$0.98*10^{-3}$
**VHH$_{QM}$7**	4.88	$1.62*10^6$	$7.92*10^{-3}$
**VHH$_{QM}$9**	0.36	$1.27*10^6$	$0.46*10^{-3}$
**Megabody 3**	15.1	$0.35*10^6$	$5.34*10^{-3}$

	K_D [nM]	k_{off} [$M^{-1}s^{-1}$]	k_{on} [s^{-1}]
**VHH$_{QM}$4**	26.8	$0.14*10^6$	$3.68*10^{-3}$

Supplementary Table 3 | Raw data for Fig. 6q.

QM	wt	wt	wt	wt	wt	wt	wt	R484E	R30E	S356Y	E429R	D521A	D304A
P-AF647	wt	wt	wt	wt	D58R	S60R	E127R	wt	wt	wt	wt	wt	wt
Neu5Ac	+	-	+	+	+	+	+	+	+	+	+	+	+
VHHQM3	-	+	-	-	-	-	-	-	-	-	-	-	-
Sample 1	6984	4713	1289	348	723	1646	389	92	643	485	25	7584	4453
Sample 2	7935	2219	227	418	126	2136	423	774	397	301	30	7552	5892
Sample 3	6364	3730	638	239	739	2321	2082	401	506	1835	134	6851	6731
Total	21283	10662	2154	1005	1588	6103	2894	1267	1546	2621	189	21987	17076
Total interaction/s	23,65	11,85	2,39	1,12	1,76	6,78	3,22	1,41	1,72	2,91	0,21	24,43	18,97
Normed total interaction/s	1,00	0,50	0,10	0,05	0,07	0,29	0,14	0,06	0,07	0,12	0,01	1,03	0,80
SD	0,08	0,13	0,05	0,01	0,04	0,04	0,10	0,03	0,01	0,09	0,01	0,04	0,12
p Value		0,00724631	0,00031997	0,00012462	0,0001934	0,00053358	0,00052699	0,00017892	0,00014167	0,0007284	0,00010523	0,67266556	0,15723476

Supplementary Table 4 | Sequences of used primers.

Primer	Sequence
HiSiaQM K273C	forward: AGGTGGGATTACCGAACGTATTTTTAATTTTGCTTGTGCCTTACTC<br>reverse: CCATTCCTCCTGTGTAATGACCGAGTAAGGCACAAGCAAAAT
HiSiaQM E235C	forward: ATGACACGTTGGAACGTCGTAAATGCCGCAACTTGTAAATTAGTC<br>reverse: GTAATGGGAAGCTGTCTAGGCTATAGACTAATTTACAAGTTGCGG
Amplification VHH for cloning in pET28a	forward (+pelB): GATCCGGGATCCATGAAATACCTATTGCCTACGGC<br>forward (-pelB): GATCCGGGATCCATGCAGGTGCAGCTCGTGGA<br>reverse: CAGTACCTCGAGTGAGGAGACGGTGACCTG
Amplification VHH with SapI for megabody	forward: GATCCTGCTCTTCCGGGTCTCTGAGACTCTCCT<br>reverse: CCTGCAGCTCTTCTTGGGTCCCCTGGCCC
MSP1D1-H5 deletion primer	forward: GGAACCGAGCCCATTAGGCGAAGAAATGCGCGATC<br>reverse: TAATGGGCTCGGTTCCACCTTTTGACGATATAATTCCATCTC
HiSiaPQM R127A$_{P}$	forward: GGAACTGCCCAAACGACTTCAAATCGTGCAATCAACAGTAT<br>reverse: TCGTTTGGGCAGTTCCGTTATAAGCTTGGGAAAGTAAAGTC
HiSiaPQM R147A$_{P}$	forward: CTTAAAACTTGCTGTGCCAAATGCAGCAACAAACTTAGCCTA<br>reverse: GGCACAGCAAGTTTTAAGCCTTTCATATCTGCAATACTGTTG
HiSiaPQM F74S$_{P}$	forward: CAGCTGAGTTACCCTGAAGCGGCAGTATTTGCCTTAC<br>reverse: CAGGGTAACTCAGCTGGAAGCGAGCAGATTCTGCAAA
HiSiaPQM L114S$_{P}$	forward: GATAAAGATAGTGGCGTGACTTTACTTTCCCAAGCTTATAACG<br>reverse: CACGCCACTATCTTTATCCATTTTTTTAATTAAATCTTTACCGAATTCTG
HiSiaPQM F111A$_{Q}$	forward: GCATCAGCCCCTATTGATGCCTTAGGAGGCATTTCTGAAAAA<br>reverse: CAATAGGGGCTGATGCACCGTTAAAAGTACGAATACCGAAATG
HiSiaPQM D114K$_{Q}$	forward: CCTAAGGCCTTAATAGGGAATGATGCACCGTTAAAAGTACGAAT<br>reverse: CCCTATTAAGGCCTTAGGAGGCATTTCTGAAAAATGGATTTTCG
HiSiaPQM Y41A$_{P}$	forward: AACTTGGAGCAAGTGAAATTTCAATTTTACCCTGTGATTTTTCTTTG<br>reverse: AATTTCACTTGCTCCAAGTTCACAATTAGGTGATGACCGTG
HiSiaPQM Q45A$_{P}$	forward: CAAGTTCAGCATTAGGTGATGACCGTGCAATGTTAAAACAATTAAAAG<br>reverse: CACCTAATGCTGAACTTGGATAAAGTGAAATTTCAATTTTACCCTGTG
HiSiaPQM Q45R$_{P}$	forward: CAAGTTCAAGGTTAGGTGATGACCGTGCAATGTTAAAACAATTAAAAG<br>reverse: CACCTAACCTTGAACTTGGATAAAGTGAAATTTCAATTTTACCCTGTG
HiSiaPQM Q55R$_{P}$	forward: CAATGTTAAAAAGATTAAAAGACGGTTCTCTCGACTTTACCTTTGCAG<br>reverse: CGTCTTTTAATCTTTTTAACATTGCACGGTCATCACCTAATTGTGAAC
HiSiaPQM D58N$_{P}$	forward: CAATTAAAAAACGGTTCTCTCGACTTTACCTTTGCAGAATCTGCT<br>reverse: GAGAGAACCGTTTTTTAATTGTTTTAACATTGCACGGTCATCAC
HiSiaPQM D58R$_{P}$	forward: CAATTAAAAAGAGGTTCTCTCGACTTTACCTTTGCAGAATCTGCT<br>reverse: GAGAGAACCTCTTTTTAATTGTTTTAACATTGCACGGTCATCACC
HiSiaPQM S60R$_{P}$	forward: AGACGGTAGACTCGACTTTACCTTTGCAGAATCTGCTC<br>reverse: GTCGAGTCTACCGTCTTTTAATTGTTTTAACATTGCACGGTC
HiSiaPQM E225Q$_{P}$	forward: GACTTATAAACAACTCCCTGAAGATCTTCAAAAAGTCGTAAAAGATGC<br>reverse: CAGGGAGTTGTTTATAAGTCTCGTTGCTTACTAAATAAAGTTGGTCAT
HiSiaPQM W227R$_{M}$	forward: GACACGTAGGAACGTCGTAAATGCCGCAACTGAAAAATTAG<br>reverse: GACGTTCCTACGTGTCATAGAAAAATAAAGTAGAGTAGCAATAAATAAG
HiSiaPQM E235R$_{M}$	forward: CCGCAACTAGAAAATTAGTCTATAGCCTAGACAGCTTCCCATTAC<br>reverse: GACTAATTTTCTAGTTGCGGCATTTACGACGTTCCAACGTGTC
HiSiaPQM R484E$_{M}$	forward: CTTGTTCCTCCGCAATCATATCGCCAAAGAAAGTCACGGTC<br>reverse: GATTGCGGAGGAACAAGTCGCAATGCGTGTTGCTGATGT
HiSiaPQM Q486A$_{M}$	forward: CGTGAAGCAGTCGCAATGCGTGTTGCTGATGTGTTTGTT<br>reverse: TTGCGACTGCTTCACGCGCAATCATATCGCCAAAGAAAG
HiSiaPQM E172R$_{P}$	forward: GCATTTTCTAGAGTTTATCTTGCGTTACAAACCAATGCCGTCG<br>reverse: CAAGATAAACTCTAGAAAATGCCATTGGTGTTGGTGATGCACC
HiSiaPQM Q178A$_{P}$	forward: GCGTTAGCAACCAATGCCGTCGATGGTCAAGAAAACCC

	reverse: GCATTGGTTGCTAACGCAAGATAAACTTCAGAAAATGCCATTGG
HiSiaPQM N150D$_P$	forward: GTGTGCCAGATGCAGCAACAAACTTAGCCTATGCTAAATATGTTG<br>reverse: GCTGCATCTGGCACACGAAGTTTTAAGCCTTTCATATCTGCAATA
HiSiaPQM R30E$_Q$	forward: GAAAAACTTGCTCTGAAAGAATTTGAGCGATAAGAATACCGAAAATG<br>reverse: TTCTTTCAGAGCAAGTTTTTCATTCTCCGTTAATTTGGAGTGAAGAA
HiSiaPQM E41Q$_Q$	forward: GCGAGTTCTTGACTCCAAATTAACGGAGAATGAAAAACTTGGC<br>reverse: GGAGTCAAGAACTCGCCAAGCTCTTATTTGTTTACGTGGG
HiSiaPQM E42Q$_Q$	forward: GCGAGTTGTTCACTCCAAATTAACGGAGAATGAAAAACTTGGC<br>reverse: GGAGTGAACAACTCGCCAAGCTCTTATTTGTTTACGTGGG
HiSiaPQM E41Q$_Q$/E42Q$_Q$	forward: GCGAGTTGTTGACTCCAAATTAACGGAGAATGAAAAACTTGGC<br>reverse: GGAGTCAACAACTCGCCAAGCTCTTATTTGTTTACGTGGG
HiSiaPQM N354Y$_M$	forward: ATAGATTCGTAGGCAATTACACCGTAAATAATCATTGCAATACTT<br>reverse: TAATTGCCTACGAATCTATCGCAAAACTCTTTATTGCAGGTTTTAT
HiSiaPQM S356Y$_M$	forward: GCCAATGAATATATCGCAAAACTCTTTATTGCAGGTTTTATTCCAG<br>reverse: TTTGCGATATATTCATTGGCAATTACACCGTAAATAATCATTGCAATAC
HiSiaPQM E429R$_M$	forward: GTCCAACAAGATCTGCCATTGTTGCAGCAGCATACTCTG<br>reverse: GGCAGATCTTGTTGGACTGAATAAGCCTGAAAAAATACCACC
HiSiaPQM S298A$_M$	forward: CTGGTATGGCAGGTTCAGCACTTGCTGATGCGGG<br>reverse: GAACCTGCCATACCAGAGAACAATAAACTTGCGCCGATATT
HiSiaPQM D304A$_M$	forward: CTTGCTGCTGCGGGGGGCTTAGGTCAATTGGAAATC<br>reverse: CCCGCAGCAGCAAGTGCTGAACCTGACATACCAGA
HiSiaPQM D521A$_M$	forward: GTTCATTGCTGCCCTAGCATTACAATTTTTAGTATTACCAATGCTTATT<br>reverse: CTAGGGCAGCAATGAACATTCCAAGAAAAAGTAACAGTGCGT
HiSiaP K254C	forward: CAAAATATCACACTAAATTATTCGTAGATGGAGAGTGTGATTTAGTC<br>reverse: CACGCCTTGTTTTTCAAAGAATGTGACTAAATCACACTCTCC
VHH$_{QM}$3 S85C	forward: CAAATGAACTGCCTGAAACCTGAGGACACAGCCGTTTATTAC<br>reverse: GTTTCAGGCAGTTCATTTGAAGATACACCGTGTTCTTGGCGT

Supplementary Table 5 | Sequences used for the conservation plot in Supplementary Fig. 15

**Q-domain**	**residues**
NMPREF0198_0032-SiaQ	1-152
NT05HA_0543-SiaQ	1-153
FN1473-SiaQ	1-153
GGC_1730-SiaQ	1-153
SiaQ-HI	1-153
UMN179_02202-SiaQ	1-156
Trebr_2165-SiaQ	1-157
PM1708-SiaQ	1-160
VV2_0733-SiaQ	1-168
PCNPT3_02946-SiaQ	1-169
P3TCK_01175-SiaQ	1-170
VPMS16_3066-SiaQ	1-173
VC1779-SiaQ	1-173
**M-domain**	**residues**
Trebr_2166-SiaM	1-427
P3TCK_01175-SiaM	1-427
VV2_0733-SiaM	1-427
VPMS16_3066-SiaM	1-427
PCNPT3_02946-SiaM	1-427
VC1779-SiaM	1-427
NMPREF0198_0032-SiaM	1-429
PM1708-SiaM	1-429
SiaM-HI	1-429
GGC_1730-SiaM	1-429
NT05HA_0543-SiaM	1-429
FN1473-SiaM	1-430
UMN179_02202-SiaM	1-430

Supplementary Table 6 | Sequences used for the conservation plot in Supplementary Fig. 16

Q-domain	residues
N1258-SiaQ	1-147
EFER_1530-SiaQ	1-158
Clobol_03198-SiaQ	1-158
Bpro_3107-SiaQ	1-162
BB2442-SiaQ	1-163
Asuc_0158-SiaQ	1-163
Bpro_1871-SiaQ	1-164
NAS141_03721-SiaQ	1-164
RD1_1052-SiaQ	1-164
HICG_00826-SiaQ	1-165
Rfer_1840-SiaQ	1-165
Dde_0634-SiaQ	1-165
Desal_0342-SiaQ	1-165
BB3421-SiaQ	1-166
NAS141_03681-SiaQ	1-169
Csal_0660-SiaQ	1-169
pro_4736-SiaQ	1-172
SM_b20295-SiaQ	1-172
Desal_2161-SiaQ	1-172
Smed_3836-SiaQ	1-172
CPS_0129-SiaQ	1-173
Oant_3902-SiaQ	1-173
BB0719-SiaQ	1-174
Dde_1548-SiaQ	1-174
RD1_3994-SiaQ	1-175
Csal_2479-SiaQ	1-177
Veis_3954-SiaQ	1-178
RD1_0742-SiaQ	1-180
Oant_4429-SiaQ	1-180
BBta_0128-SiaQ	1-181
Apre_1383-SiaQ	1-183
MIM_c39430-SiaQ	1-185

RPB_3329-SiaQ	1-186
Xaut_3368-SiaQ	1-187
Sdel_0447-SiaQ	1-187
Bpro_0088-SiaQ	1-188
SM_b20442-SiaQ	1-190
Atu3253-SiaQ	1-190
bll6834-SiaQ	1-192
SPO1773-SiaQ	1-192
Bamb_6123-SiaQ	1-192
Csal_0678-SiaQ	1-198
H16_A1328-SiaQ	1-200
RPB_2686-SiaQ	1-201
Shew_1446-SiaQ	1-212
SO_3134-SiaQ	1-219
**M-domain**	**residues**
SPO1773-SiaM	1-419
HICG_00826-SiaM	1-423
Oant_3902-SiaM	1-424
Bpro_1871-SiaM	1-425
Veis_3954-SiaM	1-425
BB3421-SiaM	1-425
Desal_2161-SiaM	1-425
Oant_4429-SiaM	1-425
RD1_1052-SiaM	1-425
Xaut_3368-SiaM	1-426
CPS_0129-SiaM	1-426
RPB_2686-SiaM	1-426
MIM_c39430-SiaM	1-426
Desal_0342-SiaM	1-426
NAS141_03681-SiaM	1-426
Smed_3836-SiaM	1-426
SM_b20295-SiaM	1-426
Csal_0660-SiaM	1-426
Csal_2479-SiaM	1-426
RD1_3994-SiaM	1-427

Dde_1548-SiaM	1-427
Sdel_0447-SiaM	1-427
FN1258-SiaM	1-428
Bpro_3107-SiaM	1-429
Asuc_0158-SiaM	1-430
BB0719-SiaM	1-430
BB2442-SiaM	1-430
BBta_0128-SiaM	1-430
RPB_3329-SiaM	1-430
RD1_0742-SiaM	1-431
Dde_0634-SiaM	1-433
Apre_1383-SiaM	1-433
H16-A1328-SiaM	1-434
Clobol_03198-SiaM	1-435
NAS141_03721-SiaM	1-436
Csal_0678-SiaM	1-440
Bpro_4736-SiaM	1-440
Bpro_0088-SiaM	1-446
Atu3253-SiaM	1-447
Rfer_1840-SiaM	1-449
Bamb_6123-SiaM	1-450
EFER_1530-SiaM	1-452
Shew_1446-SiaM	1-465
SO_3134-SiaM	1-465
bll6834-SiaM	1-468
SM_b20442-SiaM	1-468

Supplementary References

1. Punjani, A., Rubinstein, J. L., Fleet, D. J. & Brubaker, M. A. cryoSPARC: algorithms for rapid unsupervised cryo-EM structure determination. *Nat Methods* **14**, 290-296 (2017).
2. Scheres, S. H. W. RELION: implementation of a Bayesian approach to cryo-EM structure determination. *Journal of structural biology* **180**, 519-530 (2012).
3. Ovchinnikov, S., Kamisetty, H. & Baker, D. Robust and accurate prediction of residue–residue interactions across protein interfaces using evolutionary information. *eLife* **3**, 1061-1021 (2014).
4. Jumper, J. et al. Highly accurate protein structure prediction with AlphaFold. *Nature* **596**, 583-589 (2021).
5. Chowdhury, N. et al. The VC1777–VC1779 proteins are members of a sialic acid-specific subfamily of TRAP transporters (SiaPQM) and constitute the sole route of sialic acid uptake in the human pathogen Vibrio cholerae. *Microbiology* **158**, 2158-2167 (2012).
6. Vetting, M. W. et al. Experimental strategies for functional annotation and metabolism discovery: targeted screening of solute binding proteins and unbiased panning of metabolomes. *Biochemistry* **54**, 909-931 (2015).
7. Sievers, F. & Higgins, D. G. in *Multiple sequence alignment methods* 105-116 (Springer, 2014).
8. Waterhouse, A. M., Procter, J. B., Martin, D. M. A., Clamp, M. & Barton, G. J. Jalview Version 2—a multiple sequence alignment editor and analysis workbench. *Bioinformatics* **25**, 1189-1191 (2009).
9. Schneider, T. D. & Stephens, R. M. Sequence logos: a new way to display consensus sequences. *Nucleic Acids Res* **18**, 6097-6100 (1990).
10. Crooks, G. E., Hon, G., Chandonia, J.-M. & Brenner, S. E. WebLogo: a sequence logo generator. *Genome research* **14**, 1188-1190 (2004).
11. Williams, C. J. et al. MolProbity: More and better reference data for improved all-atom structure validation. *Protein Science* **27**, 293-315 (2018).
