# Supplementary material for: Structural and mechanistic analysis of a tripartite ATP-independent periplasmic TRAP transporter
Source: Nat Commun. 2022 Aug 4;13:4471. doi: 10.1038/s41467-022-31907-y (PMC9352664; doi:10.1038/s41467-022-31907-y)
Supplement: Supplementary file 3 — Description of Additional Supplementary Files [file 41467_2022_31907_MOESM3_ESM.pdf]

Description of Additional Supplementary Files

File name: Supplementary Data 1

Description: Model of the outward open tripartite transporter complex and a molprobity validation.

File name: Supplementary Data 2

Description: Model of the inward open tripartite transporter complex and a molprobity validation.

File name: Supplementary Movie 1

Description: **Movies showing HiSiaQM labelled with Nb3- Af555 in a SSB.** Image sequences correspond to the images shown in Fig 6 b and c. Scale bar equals 3 µm.

File name: Supplementary Movie 2

Description: **Movies showing the interactions of single AF647-labelled P-domain variants with SSBs containing HiSiaQM.** Image sequences correspond to the maximum intensity projections shown in Fig 6 d-j. Scale bar equals 3 µm.

File name: Supplementary Movie 3

Description: **Movies showing the interactions of single AF647-labelled P-domains with SSBs containing HiSiaQM variants.** Image sequences correspond to the maximum intensity projections shown in Fig 6 k-p and Fig 6d. Scale bar equals 3 µm.

File name: Supplementary Movie 4

Description: **Comparison of the raw data with resulting single molecules trajectories.** Raw data image sequence (left panel) corresponds to the maximum intensity projections shown in Fig 6 d. The middle panel shows the corresponding single molecule tracks over time by the Trackmate tracking algorithm. The right panel shows an overlay of the two image sequences.
