# Supplementary material for: Structural and mechanistic analysis of a tripartite ATP-independent periplasmic TRAP transporter
Source: Nat Commun. 2022 Aug 4;13:4471. doi: 10.1038/s41467-022-31907-y (PMC9352664; doi:10.1038/s41467-022-31907-y)
Supplement: Supplementary file 4 — Supplementary Data 1 [file 41467_2022_31907_MOESM4_ESM.zip › Molprobity report for the outward open tripartite complex model.pdf]

Analysis output: all-atom contacts and geometry for openStateComplexFH.pdb

Summary statistics

All-Atom Contacts	Clashscore, all atoms:	15.78		46th percentile* (N=1784, all resolutions)
	Clashscore is the number of serious steric overlaps (> 0.4 Å) per 1000 atoms.			
Protein Geometry	Poor rotamers	25	3.30%	Goal: <0.3%
	Favored rotamers	705	93.01%	Goal: >98%
	Ramachandran outliers	5	0.54%	Goal: <0.05%
	Ramachandran favored	897	97.71%	Goal: >98%
	Rama distribution Z-score	-0.34 ± 0.25		Goal: abs(Z score) < 2
	MolProbity score^	2.16		67th percentile* (N=27675, 0Å - 99Å)
	Cβ deviations >0.25Å	5	0.58%	Goal: 0
	Bad bonds:	11 / 7312	0.15%	Goal: 0%
	Bad angles:	12 / 9913	0.12%	Goal: <0.1%
Peptide Omegas	Cis Prolines:	2 / 39	5.13%	Expected: ≤1 per chain, or ≤5%
	Twisted Peptides:	2 / 921	0.22%	Goal: 0
Low-resolution Criteria	CaBLAM outliers	5	0.5%	Goal: <1.0%
	CA Geometry outliers	0	0.00%	Goal: <0.5%
Additional validations	Tetrahedral geometry outliers	1		
	Waters with clashes	0/0	0.00%	See UnDowser table for details

In the two column results, the left column gives the raw count, right column gives the percentage.
* 100th percentile is the best among structures of comparable resolution; 0th percentile is the worst. For clashscore the comparative set of structures was selected in 2004, for MolProbity score in 2006.
^ MolProbity score combines the clashscore, rotamer, and Ramachandran evaluations into a single score, normalized to be on the same scale as X-ray resolution.
Key to table colors and cutoffs here:

By adding H to this model and allowing Asn/Gln/His flips, you have already fixed 1 bad rotamer and improved your clashscore by 0.48 points.
Make sure you download the modified PDB to take advantage of these improvements!
NOTE: Atom positions have changed, so refinement to idealize geometry is necessary.

Multi-criterion visualizations

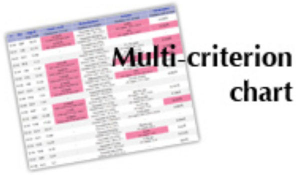

View (1.1 Mb)

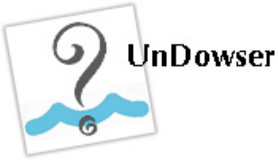

View (2.8 Kb)

Single-criterion visualizations

- **Clash list** (9.6 Kb): View
- **Ramachandran plot kinemage** (466 Kb): View in KiNG | View in NGL | Download
- **Ramachandran plot PDF** (1.7 Mb): View
- **Ramachandran distribution Z-score analysis** (43 Kb): View
- **Chiral volume report** (806 bytes): View

- **Cβ deviation scatter plot** (44 Kb): View in KiNG | View in NGL | Download

Continue >

About MolProbity | Website for the Richardson Lab | Using ecloud x-H | Internal reference 4.5.1
