# Supplementary material for: Structural and mechanistic analysis of a tripartite ATP-independent periplasmic TRAP transporter
Source: Nat Commun. 2022 Aug 4;13:4471. doi: 10.1038/s41467-022-31907-y (PMC9352664; doi:10.1038/s41467-022-31907-y)
Supplement: Supplementary file 10 — Reporting Summary [file 41467_2022_31907_MOESM10_ESM.pdf]

nature portfolio

Corresponding author(s): Gregor Hagelueken

Last updated by author(s): Jun 22, 2022

Reporting Summary

Nature Portfolio wishes to improve the reproducibility of the work that we publish. This form provides structure for consistency and transparency in reporting. For further information on Nature Portfolio policies, see our Editorial Policies and the Editorial Policy Checklist.

Statistics

For all statistical analyses, confirm that the following items are present in the figure legend, table legend, main text, or Methods section.

n/a	Confirmed	
☐	☒	The exact sample size (*n*) for each experimental group/condition, given as a discrete number and unit of measurement
☐	☒	A statement on whether measurements were taken from distinct samples or whether the same sample was measured repeatedly
☐	☒	The statistical test(s) used AND whether they are one- or two-sided<br>*Only common tests should be described solely by name; describe more complex techniques in the Methods section.*
☒	☐	A description of all covariates tested
☒	☐	A description of any assumptions or corrections, such as tests of normality and adjustment for multiple comparisons
☐	☒	A full description of the statistical parameters including central tendency (e.g. means) or other basic estimates (e.g. regression coefficient) AND variation (e.g. standard deviation) or associated estimates of uncertainty (e.g. confidence intervals)
☐	☒	For null hypothesis testing, the test statistic (e.g. *F*, *t*, *r*) with confidence intervals, effect sizes, degrees of freedom and *P* value noted<br>*Give P values as exact values whenever suitable.*
☒	☐	For Bayesian analysis, information on the choice of priors and Markov chain Monte Carlo settings
☒	☐	For hierarchical and complex designs, identification of the appropriate level for tests and full reporting of outcomes
☒	☐	Estimates of effect sizes (e.g. Cohen's *d*, Pearson's *r*), indicating how they were calculated

Our web collection on statistics for biologists contains articles on many of the points above.

Software and code

Policy information about availability of computer code

Data collection	The cryoEM dataset was collected at the IGBMC in Strassbourg using a Krios Titan microscope from ThermoFisher
Data analysis	The cryoEM dataset was processed with cryoSPARC (3.3.1), RELION (3.1) and phenix (1.20-4444). csparc2star.py was used to migrate data from cryoSPARC to RELION (https://doi.org/10.5281/zenodo.3576630). Alphafold2 (https://github.com/deepmind/alphafold) was used for the structural predictions. Coot (0.9.6) and ChimeraX (1.3) were used for structural modelling. Fiji (1.52p) was used for image analysis.

For manuscripts utilizing custom algorithms or software that are central to the research but not yet described in published literature, software must be made available to editors and reviewers. We strongly encourage code deposition in a community repository (e.g. GitHub). See the Nature Portfolio guidelines for submitting code & software for further information.

Data

Policy information about availability of data

All manuscripts must include a data availability statement. This statement should provide the following information, where applicable:

- Accession codes, unique identifiers, or web links for publicly available datasets
- A description of any restrictions on data availability
- For clinical datasets or third party data, please ensure that the statement adheres to our policy

The coordinate and map data generated in this study have been deposited in the PDB and EMDB databases under accession code 7QE5 [http://doi.org/] and EMD-13930 [http://doi.org/]. The movie data generated in this study are provided in the Supplementary Information. Data underlying all plots are provided as Source data. Source data are provided with this paper.
The coordinate data used in this study are available in the PDB database under accession codes 5UL9 [http://doi.org/10.2210/pdb5UL9/pdb]
2CEY [http://doi.org/10.2210/pdb2CEY/pdb]

3B50 [http://doi.org/10.2210/pdb3B50/pdb]
5NVA [http://doi.org/10.2210/pdb5NVA/pdb]
2HZL [http://doi.org/10.2210/pdb2HZL/pdb]
2ZZV [http://doi.org/10.2210/pdb2ZZV/pdb])

Field-specific reporting

Please select the one below that is the best fit for your research. If you are not sure, read the appropriate sections before making your selection.

☒ Life sciences ☐ Behavioural & social sciences ☐ Ecological, evolutionary & environmental sciences

For a reference copy of the document with all sections, see nature.com/documents/nr-reporting-summary-flat.pdf

Life sciences study design

All studies must disclose on these points even when the disclosure is negative.

Sample size	No sample size calculation was performed. Biochemical experiments were performed in triplicate. Exceptions are the VHH SPR experiments, since they were only needed to select the strongest binding VHHs for downstream experiments. The binding of the VHHs was independently supported by the in vivo experiments, the cryo-EM structure and the competition experiment between VHHQM3 and HiSiaP.
Data exclusions	No data was excluded.
Replication	The ITC binding experiments, the in vivo experiments and microscopy experiments were performed in triplicate. Four SPR experiments werre performed to study the interaction of the P- and QM-domains. The replication was successful.
Randomization	Randomization was not performed since it is not applicable to this type of study.
Blinding	Blinding was not performed, since it is not applicable to this type of study.

Reporting for specific materials, systems and methods

We require information from authors about some types of materials, experimental systems and methods used in many studies. Here, indicate whether each material, system or method listed is relevant to your study. If you are not sure if a list item applies to your research, read the appropriate section before selecting a response.

Materials & experimental systems

n/a	Involved in the study	
☐	☒	Antibodies
☒	☐	Eukaryotic cell lines
☒	☐	Palaeontology and archaeology
☐	☒	Animals and other organisms
☒	☐	Human research participants
☒	☐	Clinical data
☒	☐	Dual use research of concern

Methods

n/a	Involved in the study	
☒	☐	ChIP-seq
☒	☐	Flow cytometry
☒	☐	MRI-based neuroimaging

Antibodies

Antibodies used	6x-His Tag Monoclonal Antibody (4E3D10H2/E3) from Invitrogen (1:1000 dilution). A well established standard Antibody.<br>For detection: m-IgGk BP-HRP from Santa Cruz (sc-516102) (1:5000 dilution).
Validation	The 6xHis antibody was purchased from ThermoFisher (Catalog # MA1-135):<br>https://www.thermofisher.com/order/genome-database/dataSheetPdf?producttype=antibody&productsubtype=antibody_primary&productId=MA1-135&version=233<br><br>The m-IgGk BP-HRP for detection was purchased from SantaCruz. https://www.scbt.com/p/m-igg-kappa-bp-hrp

Animals and other organisms

Policy information about studies involving animals; ARRIVE guidelines recommended for reporting animal research

Laboratory animals	Male alpacas (Vicugna pacos) were used for the generation of VHHs used in this study.

Wild animals	not applicable
Field-collected samples	not applicable
Ethics oversight	All immunizations were authorized by the Landesuntersuchungsamt Rheinland-Pfalz (23 177-07/A 17-20-005 HP)

Note that full information on the approval of the study protocol must also be provided in the manuscript.
